# Pericyte Snai1 ablation prevents pathological angiogenesis in retinopathy via extracellular matrix remodelling-mediated inhibition of endothelial cell activation

**DOI:** 10.1038/s12276-026-01874-1

**Published:** 2026-10-26

**Authors:** Quan Zhang, Bojiao Tang, Yang Su, Pengfei Liu, Liang Liang, Ziyan Yang, Peiran Zhang, Chaowei Tian, Ting Wen, Yanyan Duan, Danni Jia, Yue Zhou, Guorui Dou, Hua Han, Yusheng Wang, Jiaxing Sun, Xianchun Yan

**Affiliations:** 1 Department of Ophthalmology, Eye Institute of Chinese PLA, Xijing Hospital, Fourth Military Medical University, Xi’an, 710032 China; 2 State Key Laboratory of Holistic Integrative Management of Gastrointestinal Cancers, Department of Biochemistry and Molecular Biology, Fourth Military Medical University, Xi’an, 710032 China; 3 Institute of Future Agriculture, Northwest Agriculture and Forestry University, Yangling, 712100 China; 4 Tangdu Innovative Institute of Science and Technology, Tangdu Hospital, Fourth Military Medical University, Xi’an, 710038 China

**Keywords:** Vascular diseases, Cell growth

## Abstract

Pathological retinal neovascularization (RNV) is the leading cause of blindness. Pericytes are crucial for vascular stability and involved in RNV, but the underlying mechanism remains unclear. This study investigates the role and mechanism of Snai1, a transcription factor involved in endothelial–mesenchymal transition, in regulating pericytes during RNV. By analysing single-cell RNA-seq data from oxygen-induced retinopathy (OIR) mice, we identified a distinct subpopulation of ‘dysfunctional’ pericytes with enriched pro-angiogenic pathways and upregulated Snai1. Genetic deletion of Snai1 in pericytes markedly reduced pathological neovascular tufts and vascular leakage, while improving retinal function in OIR mice, without affecting physiological angiogenesis. Mechanistically, Snai1 in pericytes directly bound to promoters and upregulated key extracellular matrix (ECM) genes, including collagens and the crosslinking enzyme Lox, which remodelled the perivascular ECM into a pro-angiogenic niche. Indeed, Snai1-overexpressing pericytes enhanced endothelial cell proliferation and tube formation only upon direct cell–cell contacts in vitro, an effect abolished by knocking down Col4a1 or inhibiting Lox activity with β-aminopropionitrile (BAPN). A feedforward loop was identified where hypoxic ECs secreted ANGPT2 and TGFβ1, stabilizing Snai1 protein in pericytes. Importantly, intravitreal injection of BAPN in OIR mice phenocopied the Snai1 ablation in effectively inhibiting RNV and promoting vascular normalization. In summary, Snai1 in pericytes is a master regulator of pathological angiogenesis in RNV, programming pericytes to construct a pro-angiogenic ECM through collagen synthesis and crosslinking, facilitating aberrant vascular growth via contact-dependent pericyte/ECM–endothelial-cell interactions. Targeting the Snai1/ECM axis represents a potential ‘vascular normalization’ strategy for RNV-related diseases.

**Figure:**
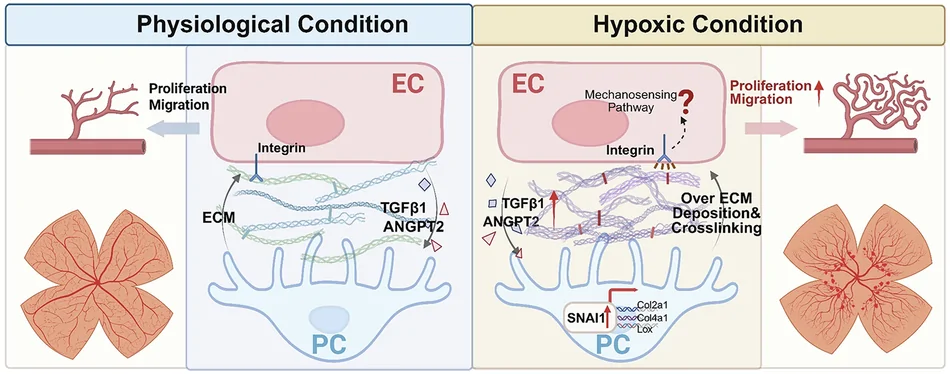

## Snai1 Drives Pathological Angiogenesis in Retinal Neovascularization

Pathological retinal neovascularization (RNV) is a key feature of retinal vascular diseases such as diabetic retinopathy, leading to fragile and haemorrhage-prone vessels. Current anti-VEGF therapies often fall short owing to poor response and recurrence. This study explores the role of pericytes and the extracellular matrix (ECM) in RNV, focusing on the transcription factor Snai1. Researchers used human samples and animal models to show that Snai1 in pericytes is crucial for collagen secretion and crosslinking, which are vital for endothelial cell activation and pathological angiogenesis. Snai1 knockout in pericytes reduced neovascularization and improved retinal function by enhancing vessel integrity. The study highlights the potential of targeting the Snai1/ECM axis to treat RNV-related diseases, suggesting that future research could focus on ECM remodelling to restore vascular homeostasis and improve therapeutic outcomes.

This summary was initially drafted using artificial intelligence, then revised and fact-checked by the author.

## Introduction

Pathological retinal neovascularization (RNV) is a hallmark feature of blindness-causing retinal vascular diseases (RVDs) such as diabetic retinopathy, retinal vein occlusion (RVO) and retinopathy of prematurity (ROP). Neovascularization arises as a compensatory response to ischaemia, but aberrant angiogenesis and remodelling in the pathological environment results in immature, fragile and haemorrhage-prone vessels with poor pericyte coverage^1^. Current clinical treatments involving anti-VEGF therapy face challenges including poor response, recurrence and unfavourable long-term outcomes^2^. Thus, elucidating the mechanisms of defective pathological neovascularization and remodelling is critical for the treatment of RNV-related diseases.

Pericytes are essential mural cells of the retinal microvasculature. They maintain vascular homeostasis by regulating lumen diameter, preserving inner blood–retinal barrier integrity, and interacting with endothelial cells via intercellular junctions and paracrine signalling. Pericytes also play a key role in vascular remodelling by restoring endothelial coverage and modifying extracellular matrix (ECM)^3,4^. Pericyte dysfunction is pivotal in RVD pathogenesis^5^: pericytes detach from capillaries under ischaemic conditions and alter their secretory profile; with disease progression, pericytes may trans-differentiate into myofibroblasts, contributing to retinal fibrosis, as indicated by recent single-cell RNA sequencing (scRNA-seq) studies. However, the pathological and molecular characteristics of pericytes in RVDs, and the mechanisms by which they regulate aberrant vascularization and remodelling, remain unclear, and effective therapeutic targets are lacking.

Another important participant in RNV is ECM. ECM is a meshwork composed of proteins, proteoglycans and glycosaminoglycans, which provides a mechanical scaffold and biochemical signals for cells^6^. Of note, collagen, as a dominant component of ECM, plays a vital role in maintaining tissue structure and facilitating cell adhesion and migration^7^. After production by various types of cells, collagen crosslinking is essential for the assembly of collagen fibrils in the extracellular space, and serves as a foremost determinant of ECM stability and mechanical stiffness^8^. Disruption of ECM components or structures significantly contributes to pathological vascular remodelling^9^. For instance, lack of crosslinking compromises vessel stability and is considered a key risk factor for aortic aneurysm^10^, whereas excessive crosslinking leads to vascular diseases such as artery stiffness and tumour angiogenesis^8^.

Recently, we identified that Snai1, a transcription factor, in endothelial cells promotes the early phase of ocular neovascularization^11^. Snai1 belongs to the Snail family, which contains three members in vertebrates, namely, Snai1, Snai2 (Slug) and Snai3. Snai1 and Snai2 have been reported to promote endothelial–mesenchymal transition^12,13^. In addition, the Snail family in endothelial cells also regulates angiogenesis^14^. In this study, we show that Snai1 in pericytes is essential for pericyte-derived collagen secretion and crosslinking, which guarantee endothelial cell activation and pathological angiogenesis in the oxygen-induced retinopathy (OIR) model. Therefore, the Snai1/ECM axis mediates a novel pericyte–endothelial-cell communication and serves as a target for the treatment of RNV-related diseases.

## Materials and methods

### Human samples

Proliferative membranes and fibrous subretinal membranes from patients with diabetic retinopathy were obtained from the Department of Ophthalmology, Xijing Hospital, Fourth Military Medical University. All of the patients included had been diagnosed with diabetic retinopathy, macular hole or retinal detachment and were scheduled for therapeutic vitrectomy. During surgery, the vitreous humour samples were also collected, immediately placed on ice, and subsequently stored at −80 °C until analysis. Detailed clinical information for all patients is summarized in Supplementary Table 1. Written informed consent was obtained from each participant prior to surgery. All vitrectomy procedures were performed by the same experienced surgeon. The use of human samples was approved by the Ethics Committee of Xijing Hospital (approval number KY20254020-1) and was conducted in accordance with the principles of the Declaration of Helsinki.

### Animal models

C57BL/6 mice were maintained under specific pathogen-free condition. *Pdgfrb-Cre*^ERT^, *Snai1*^flox/flox^ mice were purchased from Cyagen Co., Ltd. Genotyping was determined by polymerase chain reaction (PCR) and primers are listed in Supplementary Table 2. Mice of both sexes were used. To induce Cre-mediated recombination, neonatal mice received subcutaneous injections of tamoxifen (20 mg/ml in corn oil, Sigma-Aldrich, St. Louis, MO, USA). For most experiments, a daily dose of 2.5 μl was administered from postnatal day (P) 5 to P7. In some cases, to examine effects on normal retinal vascular development, pups were injected with the same dosage for three consecutive days starting at P1. Mice were euthanized using 2% inhaled isoflurane (RWD Life Science Co., Ltd, Shenzhen, China) followed by cervical dislocation, and retinas were harvested for subsequent analysis.

The OIR model was established as described before^11^. Briefly, pups and their nursing dams were placed in a high-oxygen environment (75 ± 2%) for 5 days starting at P7 and then returned to room air. Where indicated, mice at P12 were anaesthetized with 2% isoflurane and maintained under 1.5% isoflurane. Subsequently, 1 μl of β-Aminopropionitrile (BAPN, 500 μM in sterile phosphate buffered saline (PBS); MCE, New Jersey, USA) or an equal volume of sterile PBS (vehicle control) was intravitreally injected into either eye of each pup. At P17, mice were euthanized using 2% inhaled isoflurane followed by cervical dislocation, and retinas were harvested for subsequent analysis.

The choroidal neovascularization (CNV) model was induced in 6- to 8-week-old male C57BL/6J mice as previously described^11^. Mice were anaesthetized by intraperitoneal injection of 1% sodium pentobarbital solution, and pupils were dilated with tropicamide eye drops. Under a slit-lamp microscope equipped with a coverslip as a contact lens, five laser photocoagulation spots were delivered per eye using a 532-nm frequency-doubled laser (power: 90 nW; spot size: 50 μm; duration: 100 ms). Laser burns were applied approximately 1.5- to 2-disc diameters from the optic nerve. Successful Bruch’s membrane rupture was indicated by the appearance of a bubble at the laser site. Immediately after laser photocoagulation, mice received an intravitreal injection of 1 μl of BAPN or an equal volume of PBS of the lasered eye. 7 days after laser photocoagulation and injection, the mice were sacrificed for subsequent assessments. All animal experiments were reviewed and approved by the Animal Experiment Administration Committee of the Fourth Military Medical University (approval number IACUC-20221104) and conform to the guide for Care and Use of Laboratory Animals published by the National Institute of Health.

### Fluorescein fundus angiography

Mice were anaesthetized with 2% isoflurane and maintained under 1.5% isoflurane, and pupils were dilated using atropine. A drop of gatifloxacin ophthalmic gel was applied to prevent corneal dehydration. Then, a sterile solution of sodium fluorescein (2% in PBS, 10 μl/g) was administered intraperitoneally. Sequential fundus images were captured by a small animal fundus imaging system (S-5000) immediately after injection and continuously for up to 10 min to visualize the dynamic filling of the retinal vasculature. Images were analysed quantitatively to assess vascular leakage.

### Vascular leakage assessment by dextran injection

To evaluate retinal or choroidal vascular leakage, OIR or CNV mice received an injection of FITC-conjugated dextran via different routes depending on the model. For the OIR model, mice were anaesthetized and administered 100 μl of 20 kDa FITC-dextran (10 mg/ml in PBS; MCE, New Jersey, USA) via intracardiac injection at P17. For the CNV model, mice received a tail-vein injection of 100 μl of 70 kDa FITC-dextran (10 mg/ml in PBS, MCE, New Jersey, USA) at 7 days post-laser induction. After a circulation time of 10–15 min (for OIR) or 1 day (for CNV), the eyes were enucleated and immediately fixed in 4% PFA for 1 h at 4 °C. Retinas or choroid membranes were then carefully dissected, washed with PBS, and processed for immunofluorescence staining. Whole mounts were prepared and examined under a confocal microscope to visualize vascular leakage. Extravasation of FITC-dextran was identified by the presence of diffuse fluorescent signals outside the vascular lumens. Leakage was quantified by measuring the mean fluorescence area in perivascular regions using ImageJ software.

### Electroretinography examination

Mice were dark-adapted overnight (≥ 12 h) prior to recording. All subsequent procedures were performed under dim red light to preserve retinal sensitivity. Mice pupils were dilated using 1% atropine following anaesthetization with 2% isoflurane and maintained under 1.5% isoflurane. Corneal electrodes were placed on both eyes after the application of a drop of 0.5% methylcellulose to maintain electrical contact and corneal hydration. Reference and ground needles were inserted subcutaneously in the cheek and tail, respectively. Electroretinography recordings were conducted using a vision electrophysiology system (MonPackOne). Standard protocol included scotopic (dark-adapted) responses and photopic (light-adapted) responses. In the dark-adapted full-field electroretinography, the a-wave and b-wave amplitudes reflect the functional integrity of the photoreceptor layer and the inner retinal layer, respectively. The light-adapted 3.0 electroretinography b-wave amplitude serves as a standard indicator of cone pathway function. Higher amplitude of both the a-wave and b-wave is generally associated with better retinal function.

### Cell culture and treatment

Primary mouse brain pericytes were isolated from 3- to 4-week-old mice as previously described^15^. Briefly, the cerebral cortex was dissected after removal of the meninges, placed in cold Dulbecco’s Modified Eagle’s Medium (DMEM; Invitrogen, Carlsbad, CA, USA) and minced into approximately 0.5-mm^3^ pieces. The tissue fragments were digested at 37 °C for 30 min in DMEM containing collagenase type I (1 mg/ml; Millipore Sigma, Burlington, MA, USA), DNase I (100 µg/ml; Roche, Basel, Switzerland) and dispase II (20 mg/ml; Sigma-Aldrich). The digested mixture was then centrifuged at 3000 × *g* for 15 min in a 25% bovine serum albumin (BSA)-DMEM solution to pellet the microvascular fraction. The pelleted microvascular fragments were cultured in Pericyte Medium-Mouse (PM-m, Carlsbad, CA, USA) supplemented with 2% fetal bovine serum (FBS), 1% pericyte growth supplement-mouse (PGS-m) and 1% penicillin/streptomycin (P/S) for 1 week to obtain purified PCs. Purity was confirmed by immunofluorescence staining. PCs between passages 2 and 6 were used for subsequent experiments.

The mouse brain endothelial cell line (bEnd.3) was obtained from the American Type Culture Collection (ATCC, Manassas, VA, USA) and maintained in DMEM supplemented with 10% FBS and 1% P/S. Cells were cultured at 37 °C in a humidified atmosphere containing 5% CO_2_.

For cytokine stimulation experiments, pericytes were first serum-starved for 24 h in PM-m containing 1% FBS and 1% P/S, without PGS-m. Cells were then treated with ANGPT2 (500 μg/ml, Sino Biological, Beijing, China), TGFβ1 (100 μg/ml, Sino Biological) or PDGFB (100 μg/ml, Sino Biological). RNA was extracted at 24 h and protein was collected at 48 h post-treatment. To assess protein stability, 100 μM cycloheximide (CHX, MCE) was added to the culture medium, and then the protein level was determined at different time points. To test whether ANGPT2 upregulates Snai1 via integrin α5β1, pericytes were treated with ANGPT2 with or without 1 μM ATN-161 (GlpBio, Montclair, CA, USA). Snai1 protein levels were detected at 48 h.

For Snai1 overexpression, pericytes were infected with adenovirus-expressing mouse Snai1 (oeSnai1) or control adenovirus (Ctrl, Vigene, Rockville, MD, USA) at a multiplicity of infection (MOI) of 500 according to the manufacturer’s protocol. For knockdown experiments, pericytes were transfected with 50 nM Snai1 small interfering RNA (siSnai1) or negative control small interfering RNA (siRNA) (RiboBio, Guangzhou, Guangdong, China) using the Lipofectamine 2000 reagent (Invitrogen, Carlsbad, CA, USA). Similar to knockdown Col4a1, pericytes were transfected with 50 nM siRNA to Col4a1 (siCol4a1, GenePharma, Suzhou, Jiangsu, China) or a corresponding negative control. Cells were harvested at 24 or 48 h post-transfection for further analysis.

### In vitro co-culture assays

For indirect co-culture experiments, pericyte-derived conditioned medium was prepared as follows. Following adenoviral infection or siRNA transfection, pericytes were cultured in fresh PM-m with 1% FBS for 48 h. The supernatant was then collected, filtered through a 0.22-μm membrane, and stored at −80 °C for up to 1 month. To treat bEnd.3 cells, the standard culture medium was replaced with pericyte-derived conditioned medium when bEnd.3 cells reached approximately 40% confluence. After 48 h of treatment, cells were harvested for subsequent assays.

For direct co-culture experiments, both pericytes and bEnd.3 cells were trypsinized, and the two cell types were then mixed at a 1:3 (PCs to bEnd.3) ratio in suspension and plated together for further culture followed by subsequent experiments.

### Immunostaining

For retinal whole-mount immunofluorescence, retinas were collected at P7 for retinal vascular development analysis and P17 for assessment of pathological neovascularization. Retinas were fixed in 4% PFA and then blocked/permeabilized in PBS containing 5% BSA and 0.5% Triton X-100 overnight at 4 °C. Following three 5-min PBS washes, samples were incubated with primary antibodies overnight at 4 °C, washed again and then incubated with appropriate secondary antibodies. Stained retinas were flat-mounted and imaged using a confocal laser scanning microscope (A1R, Nikon, Tokyo, Japan). Quantitative analyses were performed with Image-Pro Plus 6.0 software. For developmental assessment, the distance from the optic disc to the vascular front was quantified; and the number of vascular branches and sprouts were also measured. For pathological angiogenesis, vessel diameter was calculated. Neovascular and vaso-obliterated areas were manually outlined, and their respective ratios relative to the total retinal area were calculated as previously described^11^. Pericyte coverage was estimated as the ratio of the Pdgfrb^+^ area to the Pecam1^+^ area.

For immunofluorescence on cryosections, mouse eyeballs were processed as previously described^11^. Sections were air-dried for 2 h at room temperature, washed three times with PBS, and blocked for 2 h at room temperature in PBS containing 5% BSA and 0.5% Triton X-100. Sections were then incubated with primary antibodies overnight at 4 °C, followed by corresponding secondary antibodies for 2 h at room temperature. Nuclei were counterstained with DAPI (Sigma-Aldrich) for 10 min at room temperature before mounting. Between each incubation step, sections were washed three times for 5 min each with PBS.

For immunofluorescence staining of cultured cells, cells were fixed with 4% PFA at room temperature. After fixation, cells were permeabilized and blocked for 1 h at room temperature using PBS containing 5% BSA and 0.5% Triton X-100. Subsequently, cells were incubated with primary antibodies overnight at 4 °C. Following three washes with PBS, samples were incubated with appropriate fluorescent secondary antibodies for 2 h at room temperature. Nuclei were counterstained with DAPI before mounting with coverslips. Antibodies used are listed in Supplementary Table 3.

### Apoptosis detection by TUNEL assay

Apoptosis in retinal cryosections was determined by the TUNEL method using an In Situ Cell Death Detection Kit (Elabscience, Wuhan, Hubei, China). Briefly, sections were fixed in 4% PFA for 2 h at room temperature. Tissue permeabilization was performed by incubation with Proteinase K at 37 °C for 20 min. Sections were then equilibrated with the supplied buffer, followed by incubation with the TUNEL reaction mixture in a humidified dark chamber at 37 °C for 1 h. When required, immunostaining for specific cell-surface markers was conducted after TUNEL labelling. Nuclei were counterstained with DAPI before mounting for imaging.

### Cell-proliferation assay

Cell proliferation was evaluated using either EdU incorporation or Ki67 immunofluorescence. For EdU labelling, cells were cultured in medium containing 50 μmol/l EdU (RiboBio) for 2 h, fixed with 4% PFA, and stained using an Apollo 567 staining kit according to the manufacturer’s protocol. When required, immunostaining for surface markers was performed before nuclear counterstaining with DAPI. Alternatively, proliferation was evaluated by immunostaining with anti-Ki67 antibody (Abcam, Cambridge, UK). Images were acquired using fluorescence microscopy (NI-E, Nikon) or a confocal laser scanning microscope, and the proliferation was quantified as the percentage of the total number of cells that were EdU^+^ or Ki67^+^ cells.

### Cell-migration assay

Cell migration was assessed using a Transwell assay. For pericyte migration, 3 × 10^4^ cells in PM-m containing 1% FBS were seeded into the upper transwell chamber (Corning, NY, USA). Complete PM-m was added to the lower chamber. For bEnd.3 cell migration, 1 × 10^5^ cells in serum-free DMEM were placed in the upper chamber, and pericyte-derived conditioned medium was used as the chemoattractant. After 12 h of incubation, cells that had migrated to the lower surface of the membrane were fixed with 4% PFA, stained with 0.1% crystal violet and counted under a microscope.

### Tube-formation assay

The tube-forming capacity of bEnd.3 cells was assessed using a Matrigel-based assay as previously described^16^. Briefly, 48-well plates were pre-coated with 200 μl Matrigel (Corning) and allowed to polymerize for 30 min at 37 °C. bEnd.3 (1 × 10^5^ cells in 200 μl pericyte conditioned medium or control medium) or a direct co-culture mixture (1 × 10^5^ bEnd.3 with 3.3 × 10^4^ pericytes in serum-free DMEM) were then seeded onto the polymerized gel. After incubation at 37 °C for 2–4 h, the formation of capillary-like tubular networks was observed. Representative images were captured using a phase-contrast microscope. Quantification of tubulogenesis was performed using ImageJ software with the Angiogenesis Analyzer plugin; the total tube length, number of branches and number of loops were measured for each condition.

### Enzyme-linked immunosorbent assay

The levels of ANGPT2, TGFβ1 and PDGFB in endothelial cell culture media were measured using commercial enzyme-linked immunosorbent assay (ELISA) kits (Byabscience, Nanjing, Jiangsu, China). Meanwhile, levels of Col2a1, Col4a1 and Lox in vitreous liquid were quantified using kits from ELK (Wuhan, Hubei, China). All procedures were performed in accordance with the manufacturer’s protocol. Clinical samples, including those from 5 patients with diabetic retinopathy and 5 controls, are described in Supplementary Table 1.

### Analysis of single-cell RNA sequencing data

The scRNA-seq dataset was obtained from the publicly available dataset BioProject PRJNA864092 (ref.^17^). Bioinformatic analyses were performed using the online platform Omicshare Tools (www.omicshare.com/tools). For quality control, reads with low-quality barcodes or Unique Molecular Identifiers (UMIs) were filtered out and then aligned to the reference genome. Only reads mapped to the transcriptome and intersecting an exon at least 50% were considered for UMI counting. For expression-based filtering, cells with an unusually high number of UMIs (≥ 8,000) or a high percentage of mitochondrial gene (≥ 10%) were excluded. Additionally, cells with less than 500 or more than 4,000 detected genes were removed. Doublets were identified and removed using DoubletFinder (v2.0.3), which generates artificial doublets and calculates the proportion of artificial nearest neighbours for each cell based on principal component distances; cells were then ranked by the proportion of artificial nearest neighbours and filtered according to the expected number of doublets. After quality control and normalization, dimensionality reduction was conducted with Uniform Manifold Approximation and Projection using the Seurat tool. Cell clusters were annotated based on established marker gene expression. Pseudotime trajectory analysis was performed using Monocle2 to infer state transitions. Gene Ontology (GO) and Kyoto Encyclopedia of Genes and Genomes (KEGG) enrichment analysis were conducted to evaluate the biological significance of the observed changes. The quality control data for scRNA-seq analysis are listed in Supplementary Table 4.

### RNA sequencing

For RNA-seq analysis, pericytes were transfected with siSnai1 or negative control for 24 h. Total RNA was extracted, and RNA-seq libraries were prepared and sequenced on an Illumina NovaSeq 6000 platform. Raw sequencing data have been deposited in the Sequence Read Archive database under the accession number PRJNA1416461. Subsequent bioinformatics analyses, including GO, KEGG and gene set enrichment analysis, were conducted using the online OmicShare Tools (www.omicshare.com/tools).

### Transmission electron microscopy

Tissues were obtained and fixed overnight at 4 °C in a solution containing 2.5% glutaraldehyde and 4% PFA in 0.1 M phosphate buffer. Following fixation, samples were dehydrated through a graded series of acetone, infiltrated with Epox 812 and polymerized in blocks. Ultrathin sections were cut using a diamond blade, stained with uranyl acetate and lead citrate, and examined under a transmission electron microscope (JEM-1400, JEOL, Tokyo, Japan).

### Quantitative real-time PCR

Total RNA was extracted from pericytes or bEnd.3 cells using the TRIzol reagent (Invitrogen). Complementary DNA (cDNA) was synthesized from 500 ng total RNA using a reverse transcription kit (Takara, Kusatsu, Shiga, Japan). Quantitative PCR was performed with SYBR Premix ExTaq Kit (Takara) on an ABI QuantStudio 5 real-time PCR system (Life Technologies, Waltham, MA, USA). The gene-expression level was normalized to *Rpl13a*. Primer sequences are listed in Supplementary Table 2.

### Western blotting

Cells (pericytes or bEnd.3) were lysed using the RIPA buffer (Beyotime, Shanghai, China) containing 1 mM phenylmethylsulfonyl fluoride (Sigma-Aldrich). Protein was separated by SDS-polyacrylamide gel electrophoresis and transferred onto a polyvinylidene fluoride membrane. After blocking, membranes were incubated overnight at 4 °C with specific primary antibodies, followed by incubation with appropriate HRP-conjugated secondary antibodies for 1.5 h at room temperature. Between incubation steps, membranes were washed three times for 10 min with TBS containing 0.1% Tween-20. Protein bands were visualized using an enhanced chemiluminescence system (Clinx Science Instruments) and quantified using ImageJ2x software (Rawak Software). β-Actin, Vinculin or Gapdh were used as the loading controls. Antibody details are provided in Supplementary Table 3.

### Chromatin immunoprecipitation assay

Chromatin immunoprecipitation assay was performed using a commercial kit (Cell Signaling Technology, Danvers, MA, USA) according to the manufacturer’s protocol. Briefly, pericytes were fixed and cross-linked with 1% formaldehyde for 10 min at room temperature, and the reaction was quenched by adding glycine. After two gentle washes with ice-cold PBS, cells were lysed in PBS containing a protease inhibitor cocktail. Following nuclear isolation, chromatin was subjected to sonication to an average size of 200–500 bp. The supernatant was incubated with anti-Flag antibody (CST) or control IgG for 12 h at 4 °C. Protein G magnetic beads were then added and incubated for 2 h at 4 °C. Bead-bound complexes were eluted from the beads, reverse-cross-linked and purified. Enrichment efficiency of specific DNA fragments was quantified by quantitative real-time (qRT)-PCR. Primer sequences are listed in Supplementary Table 2.

### Statistical analysis

All quantitative data are expressed as the mean ± SD. Statistical analyses were conducted using Image-Pro Plus 6.0 (Media Cybernetics, Rockville, MD, USA) and GraphPad Prism 8 software. Differences between two groups were evaluated using unpaired Student’s *t* test; comparisons among multiple groups were conducted using one-way analysis of variance followed by post hoc tests. *P* < 0.05 was considered statistically significant.

## Results

### Transit of pericytes from a quiescent to a pro-angiogenic state in retinal neovascularization

**Fig. 1:**
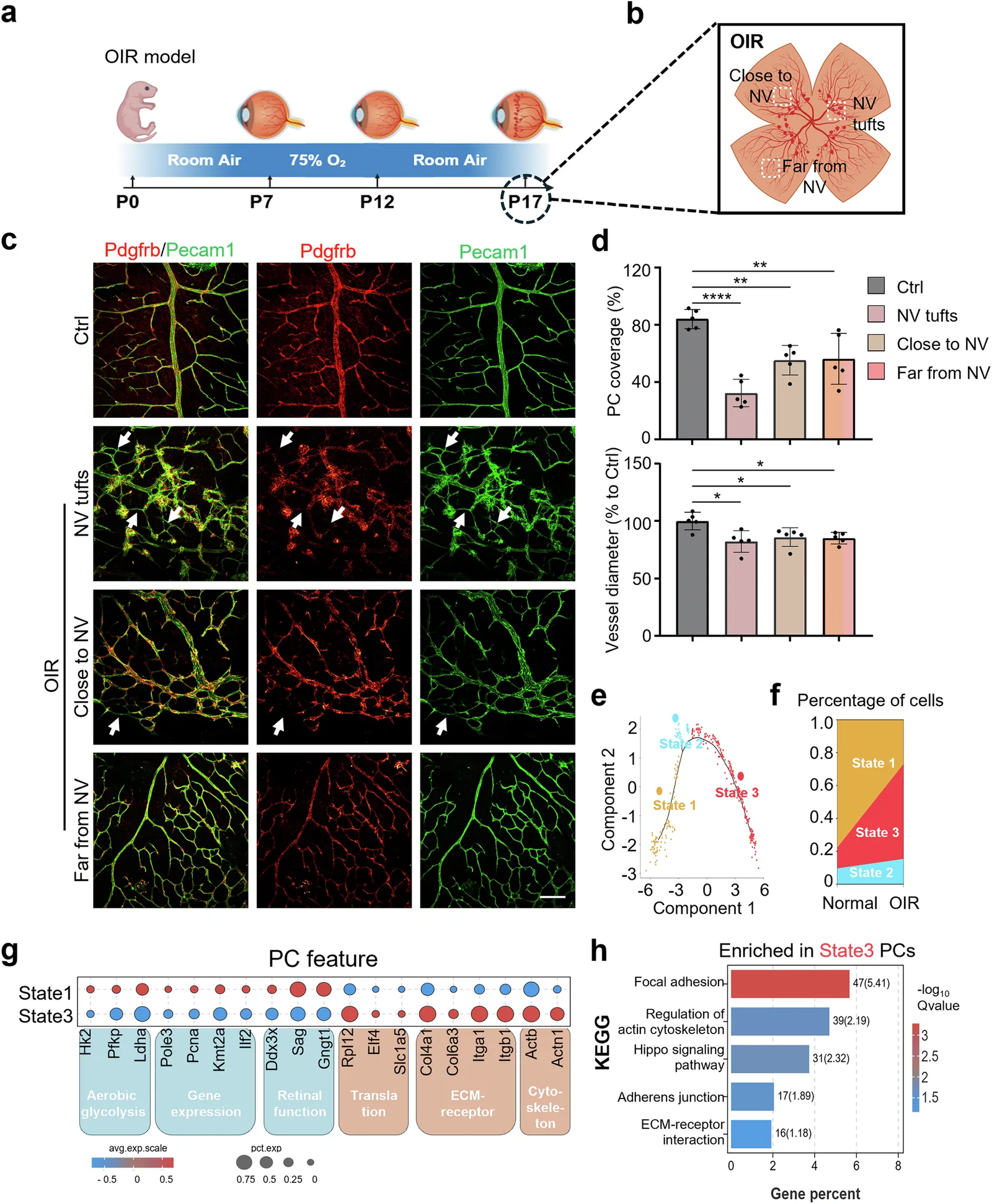
Reduction and altered differentiation states of pericytes in the murine oxygen-induced retinopathy model. **a**, **b** Schematic illustration of the oxygen-induced retinopathy (OIR) model and vascular zones in the flat-mounted retina from OIR mice. **c**, **d** Immunofluorescence of retinal flat mounts stained for Pdgfrb and Pecam1 in the control (Ctrl) and OIR mice. White arrows indicate vessels lacking pericyte (PC) coverage. The PC coverage and vascular diameter in neovascular (NV) tufts and close to or far from NV were quantitatively compared (*n* = 5). **e** Pseudotime trajectory of PCs with differentiation states generated by Monocle 2, based on single-cell RNA sequencing data from normal and OIR retinas (BioProject: PRJNA864092). **f** Proportion of PC subsets with various differentiation states in the control versus OIR retinas. **g** Characterization of molecular features and enriched genes in State1 and State3 PCs depicted with a dot plot. **h** Kyoto Encyclopedia of Genes and Genomes (KEGG) pathway enrichment analysis of upregulated genes in State3 compared with State1 PCs. Scale bar: 100 μm. Data are presented as mean ± SD. Statistical significance was determined by one-way analysis of variance with Tukey’s post hoc test for multiple comparisons (**d**). *P  <  0.05; **P  <  0.01; ****P  <  0.0001.

To investigate the role of pericytes in RNV, we used the OIR model^11^ (Fig. 1a). Retinas were harvested on P17 after the hyperoxia–normoxia treatment, and the retinal vasculature was observed by dividing into three regions: neovascular tufts, close to neovascular, and far from neovascular, after whole-mount staining (Fig. 1b). Vascular diameter and pericyte coverage were significantly reduced in OIR retinas (Fig. 1c, d). Pericyte loss was region dependent and most severe within neovascular tufts, suggesting increased migration and apoptosis at the neovascular tufts. Pericytes in OIR retinas also showed altered distribution and morphology: instead of aligning regularly along vessels, they were disorganized, often clustered at branch points, and displayed enlarged cell bodies (Supplementary Fig. 1a).

To find out critical molecules involved in pericyte alterations, we analysed scRNA-seq data from normal and OIR retinas (BioProject: PRJNA864092)^17^. Uniform Manifold Approximation and Projection analysis identified 12 retinal-cell clusters (Supplementary Fig. 1b). A total of 363 pericytes were identified using established markers (Pdgfrb, Cspg4, Kcnj8 and Des; Supplementary Fig. 1c, d) with 104 cells derived from the Ctrl group and 259 cells from the OIR group. Pseudotime trajectory analysis revealed that these pericyte subsets were connected along their differentiation route, suggesting that they represent three pericyte states (State1: 99 cells, State2: 41 cells, State3: 229 cells; Fig. 1e and Supplementary Fig. 1e). State1 was predominant in normal retinas, whereas State3 expanded in OIR (Fig. 1f), which were designated “quiescent pericytes” and “dysfunctional pericytes”, respectively, in the current study. Quiescent pericytes highly expressed genes related to aerobic glycolysis, gene-expression regulation and retina-specific functions. In contrast, dysfunctional pericytes showed elevated expression of genes involved in protein translation, ECM–receptor interaction and cytoskeletal rearrangement (Fig. 1g), which was further validated by KEGG and GO analysis (Fig. 1h and Supplementary Fig. 1f). Notably, the enrichment of “blood vessel development” in GO analysis supported a pro-angiogenic pericyte phenotype in OIR, consistent with prior reports^18^. Together, these findings suggest that pericytes undergo a pathological phenotypic transition in RNV: morphologically, from elongated and vessel aligned to contracted and clustered at branches; functionally, from vessel stabilizing to pro-angiogenic, probably through remodelling of the vascular microenvironment.

### Snai1 is selectively upregulated in pericytes during pathological retinal neovascularization

**Fig. 2:**
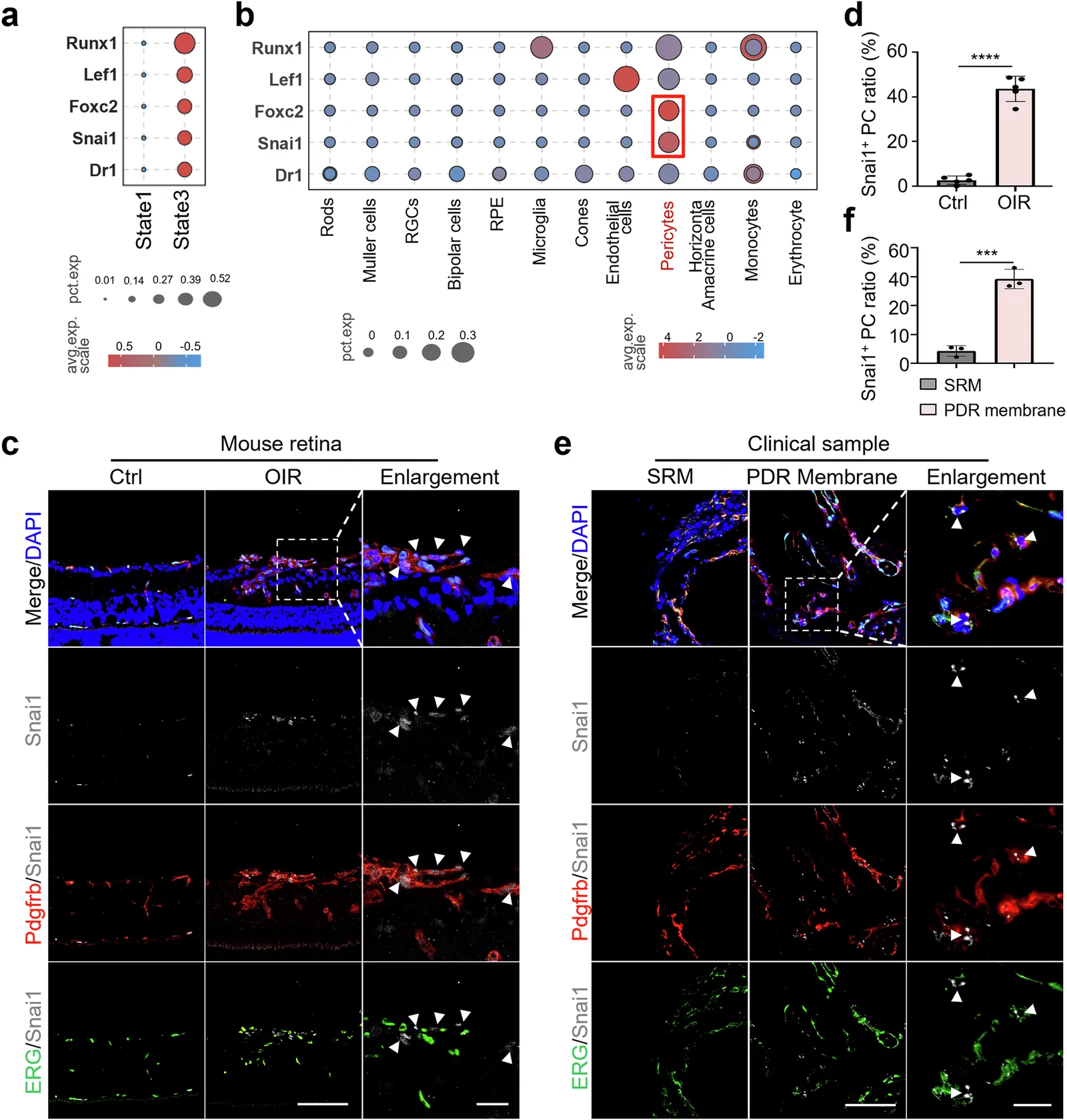
Snai1 is upregulated in pericytes during retinal neovascularization. **a**, **b** Top 5 upregulated transcription factors in State3 pericytes (PCs), and the expression of these transcription factors across different retinal-cell populations. RBCs, red blood cells; RPE, retinal pigment epithelium. **c** Immunofluorescence of retinal cryosections from control (Ctrl) and oxygen-induced retinopathy (OIR) mice at P17, stained for Pdgfrb, ERG and Snai1, with nuclei stained by DAPI. White arrowheads indicate Snai1 expression. **d** Quantification of the ratio of Snai1^+^ PCs in total PCs in the OIR and Ctrl mice on P17 (*n* = 5). **e** Immunofluorescence for Pdgfrb, ERG and Snai1 of proliferative and subretinal membranes from patients diagnosed with diabetic retinopathy. White arrowheads mark Snai1 expression. **f** Quantification of the ratio of Snai1^+^ PCs in total PCs in the proliferative and subretinal membranes (*n* = 3). PDR, proliferative diabetic retinopathy; SRM, subretinal membrane. Scale bar: 100 μm (overview); 25 μm (enlarged views). Data are presented as mean ± SD. Statistical significance was determined by unpaired Student’s *t* test (**d–f**). ***P  <  0.001; ****P  <  0.0001.

To identify critical transcription factors driving the pathological transition of pericytes, we performed differential expression analysis between quiescent and dysfunctional pericytes. The top five upregulated transcription factors in dysfunctional pericytes were Runx1, Lef1, Foxc2, Snai1 and Dr1 (Fig. 2a). Among these, Foxc2 and Snai1 exhibited specific high expression within the pericyte population (Fig. 2b). Based on our previous finding that Snai1 promotes intraocular angiogenesis by enhancing endothelial cell invasiveness, we focused on Snai1 in further investigation^11^.

Immunofluorescence staining of whole-mounted retinas showed minimal Snai1 expression in normal retinas, whereas it was markedly upregulated in the OIR group (Supplementary Fig. 2a). Snai1 was predominantly localized to neovascular tufts and co-localized with pericyte nuclei (Fig. 2c, d). However, during physiological retinal vascular development, Snai1 expression remained low through the different stages examined (Supplementary Fig. 2b, c). We further assessed Snai1 expression in human pathological tissues. In specimens from patients with diabetic retinopathy, Snai1 was detected at significantly higher levels in diabetic retinopathy proliferative membranes than in the largely avascular, fibrous subretinal membranes (Fig. 2e, f). Collectively, these results demonstrate a specific upregulation of Snai1 in pericytes under pathological neovascularization, implying its participation in pericyte dysfunction and disease-related angiogenesis.

### Pericyte-specific Snai1 knockout inhibits aberrant angiogenesis and promotes functional vascular recovery

**Fig. 3:**
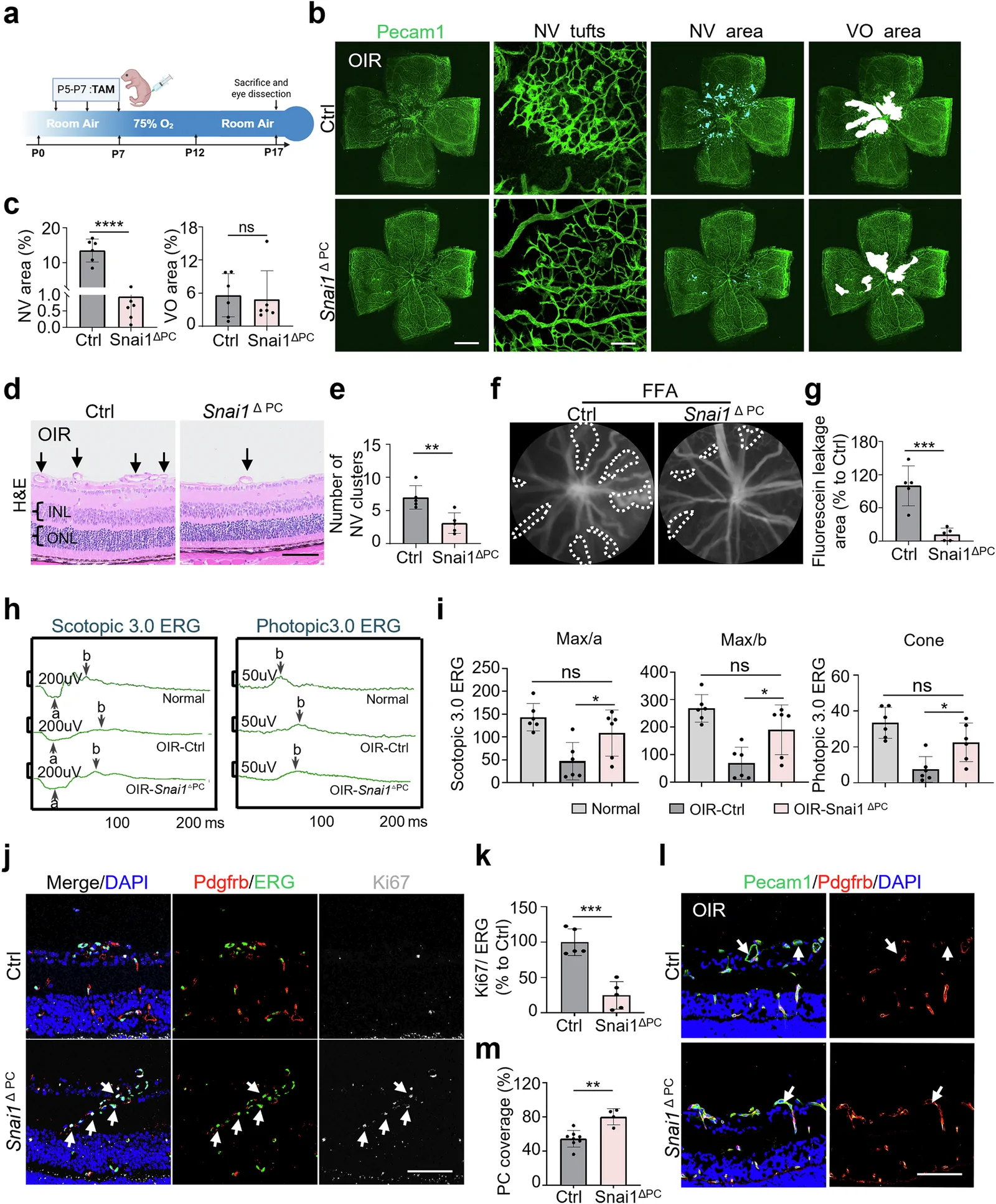
Pericyte-specific Snai1 knockout alleviates pathological neovascularization and restores vascular function in oxygen-induced retinopathy mice. **a** Experimental procedure of the oxygen-induced retinopathy (OIR) model using pericyte (PC)-specific Snai1 KO (*Snai1*^ΔPC^) and control (Ctrl) mice. Pups received daily tamoxifen injection from P5 to P7 before hyperoxia exposure. **b** Retinal flat-mounts stained for Pecam1 in the Ctrl and *Snai1*^△PC^ OIR mice at P17. Blue: neovascular (NV) tufts; white: vaso-obliteration (VO) area. **c** Quantification of NV and VO areas (*n* = 5). **d** Haematoxylin and eosin (H&E) staining of retinas, with NV cell clusters marked by arrows in the Ctrl and *Snai1*^△PC^ mice. **e** Quantification of the number of NV clusters (*n* = 5). **f** Fundus fluorescein angiography (FFA) of fundus vessels of mice. Dashed lines outline areas of capillary leakage. **g** Quantified leakage area normalized to Ctrl (*n* = 5). **h** Representative electroretinography waveforms from the normal, OIR-Ctrl and OIR-*Snai1*^ΔPC^ mice. **i** Quantification of amplitudes of waves marked in **h** (*n* = 6). **j** Retinal sections stained for Pecam1, ERG and Ki67. Arrows mark Ki67^+^ endothelial cells (ECs). **k** Percentage of Ki67^+^ERG^+^ in total ERG^+^ cells compared to control group (*n* = 5). **l** Retinal sections stained for Pecam1 and Pdgfrb. Arrows indicate vessels lacking PC coverage. **m** Percentage of PC coverage shown by the ratio of Pdgfrb^+^ area to Pecam1^+^ area (*n* = 5). Scale bar: 1 cm (overview, **b**) and 100 μm (magnified view in **b**, **d**, **j** and **l**). Data are presented as mean ± SD. Statistical significance was determined by two-tailed Student’s *t* test (**c**, **e**, **g**, **k** and **m**) and one-way analysis of variance with Tukey’s post hoc test (**i**). *P  <  0.05; **P  <  0.01; ***P  <  0.001; ****P  <  0.0001; ns not significant.

Given the specific upregulation of Snai1 in pericytes during RNV, we investigated its role using tamoxifen-inducible, pericyte-specific knockout mice (*Pdgfrb-Cre*^*ERT*^*-Snai1*^fl/fl^; *Snai1*^ΔPC^) and used *Snai1*^fl/fl^ mice as controls (Supplementary Fig. 3a, b). Retinal vascular development proceeds radially after birth, forming a complete superficial layer by P7 (ref.^19^). Analysis at P7 revealed that conditional Snai1 knockout in pericytes had minimal effects on developmental vascular outgrowth, morphology or pericyte coverage (Supplementary Fig. 3c–e). However, in the OIR model, *Snai1*^ΔPC^ mice exhibited a significant reduction in the neovascular area at P17, whereas the vaso-obliterated area remained unchanged (Fig. 3a–e), indicating that pericyte-expressed Snai1 promotes pathological angiogenesis. Furthermore, functional assessment by fluorescein angiography and dextran (20 kDa) injection revealed reduced vascular leakage in *Snai1*^ΔPC^ mice, suggesting improved vessel integrity (Fig. 3f, g and Supplementary Fig. 3f, g). Full-field electroretinography under dark-adapted and light-adapted conditions showed that Snai1 deletion in pericytes significantly increased the amplitudes of both a-waves (photoreceptor function) and b-waves (inner retinal or cone cell function) to near-normal levels, indicating a substantial recovery of neural–retinal activity (Fig. 3h, i).

To explore the mechanism underlying the attenuated angiogenesis, we assessed endothelial cell behaviour. Immunofluorescence staining indicated that the reduction in neovascularization was associated with decreased endothelial cell proliferation (Fig. 3j, k), rather than altered endothelial cell apoptosis (Supplementary Fig. 3h, i). Furthermore, Snai1 knockout in pericytes significantly improved pericyte coverage (Fig. 3l, m), contributing to the recovery of vascular functions. Together, these findings demonstrate that Snai1 deficiency in pericytes effectively suppresses pathological neovascularization and promotes the recovery and normalization of retinal vascular structure and function.

### Snai1 in pericytes promotes endothelial cell proliferation and angiogenesis through a contact-dependent mechanism

To assess the Snai1 function in pericytes, we isolated primary mouse brain microvascular pericytes and modulated Snai1 expression via adenovirus-mediated overexpression or siRNA knockdown (Supplementary Fig. 4a–e). Altering Snai1 level did not markedly affect pericyte proliferation or migration (Supplementary Fig. 5a–d). Pericytes stand in synthetic or contractile states under pathological conditions^20^ (Supplementary Fig. 5e); however, Snai1 manipulation did not significantly alter the expression of classical contraction- or synthetic-related genes (Supplementary Fig. 5f–h), suggesting that other roles are involved.

**Fig. 4:**
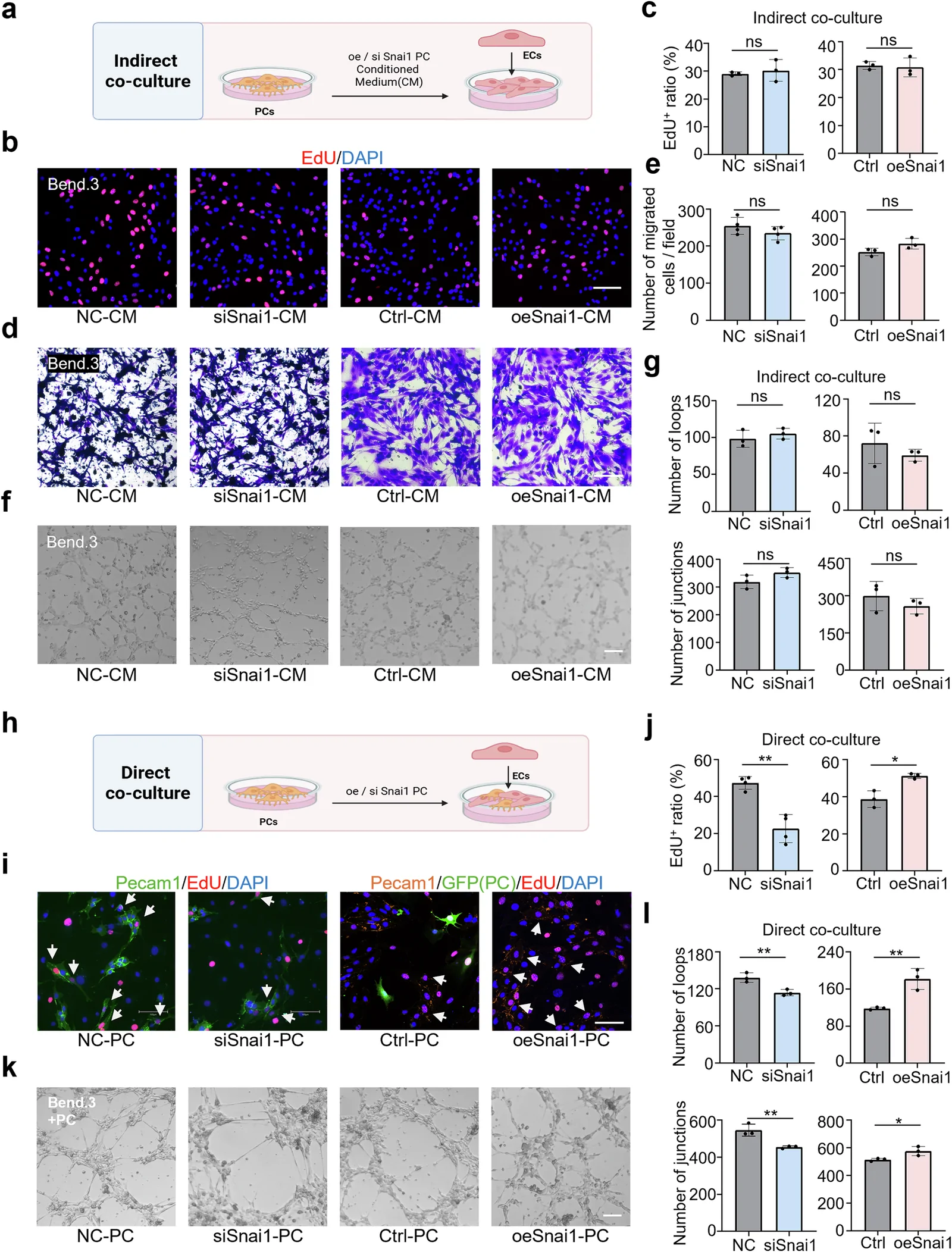
Snai1 overexpression in pericytes promotes endothelial-cell angiogenic behaviours through a contact-dependent mechanism. **a** Schematic illustration of the indirect co-culture model. **b** EdU staining of proliferating bEnd.3 cells. **c** Quantification of EdU^+^ bEnd.3 cells (*n* = 3). **d** Migration assay of bEnd.3 cells. **e** Number of migrating bEnd.3 cells (*n* = 4). **f** Tube-formation assay of bEnd.3. **g** Quantification of loops and junctions in **f** (*n* = 3). **h** Schematic illustration of the direct co-culture model. **i** Immunofluorescence staining for Pecam1 and EdU in directly co-cultured cells, with positive endothelial cells (ECs) indicated by white arrows. **j** Quantification of EdU^+^ bEnd.3 cells (*n* = 3). **k** Tube formation assay of bEnd.3-pericyte (PC) mixtures. **l** Quantification of loops and junctions in **j** (*n* = 3). Scale bar: 100 μm (**b**, **d**, **f**, **i** and **k**). Data are presented as mean ± SD. Statistical significance was determined by two-tailed Student’s *t* test (**c**, **e**, **g**, **j** and **l**). *P  <  0.05; **P  <  0.01; ns not significant.

Pericytes play a pivotal role in regulating endothelial cell activation^21^. We therefore examined whether pericyte Snai1 influences endothelial cell behaviour in vitro. In an indirect co-culture system with conditioned medium from pericytes with modulated Snai1 expression (Fig. 4a), endothelial cell migration, proliferation and tube formation were unaffected (Fig. 4b–g). By contrast, in a direct co-culture system where pericytes and endothelial cells were mixed and cultured (Fig. 4h), Snai1-overexpressing pericytes strongly enhanced endothelial cell proliferation and tube formation, whereas Snai1-knockdown pericytes attenuated endothelial cell activation (Fig. 4i–l). Together, these results indicate that Snai1 does not substantially alter pericyte proliferation, migration or phenotypic transition, but promotes endothelial cell angiogenic activity specifically through a contact-dependent mechanism.

### Pericyte Snai1 drives angiogenic extracellular matrix remodelling through collagen synthesis and crosslinking

To elucidate how Snai1 in pericytes regulates endothelial cells, we first divided pericytes into Snai1^high^ and Snai1^low^ subpopulations using the scRNA-seq data (BioProject: PRJNA864092; Snai1^high^: 85 cells; Snai1^low^: 278 cells). Bioinformatics analysis (GO, KEGG and gene set enrichment analysis) indicated that Snai1^high^ pericytes were highly enriched for pathways related to ECM organization and remodelling (Supplementary Fig. 6a–c). Because the ECM is a critical scaffold and signalling interface within the vascular niche^22^, we hypothesized that Snai1-driven ECM remodelling might modulate the pericyte–endothelial-cell crosstalk. Cell–cell communication analysis revealed that Snai1^high^ pericytes exhibited significantly more and stronger interactions with endothelial cells than Snai1^low^ pericytes (Supplementary Fig. 6d). These enhanced interactions were predominantly mediated by ECM-related ligand–receptor pairs, such as collagens and their integrin receptors (Supplementary Fig. 6e).

**Fig. 5:**
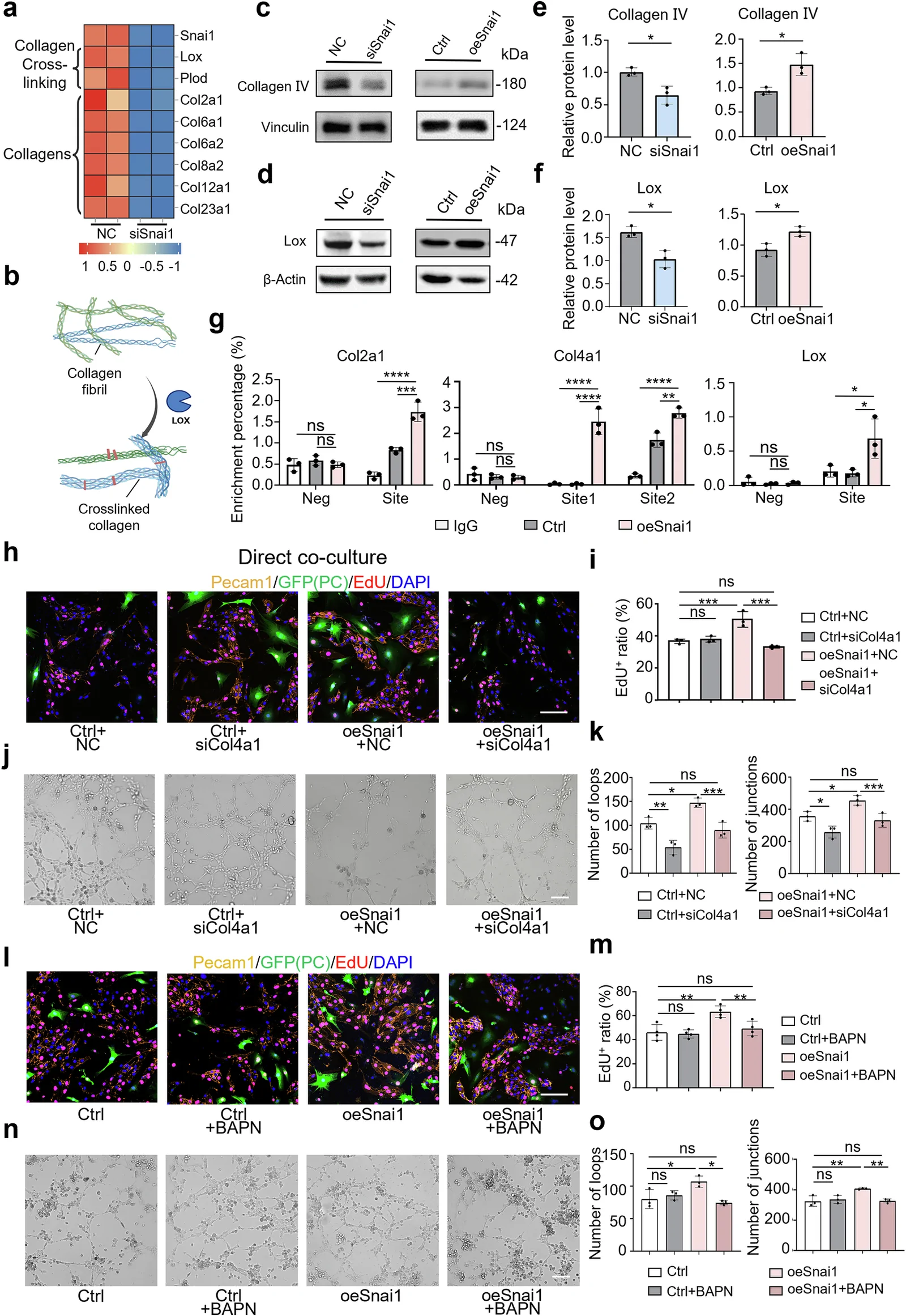
Snai1 regulates pericyte–endothelial cell crosstalk through extracellular matrix remodelling. **a** Heatmap of collagen crosslinking genes in pericyte (PCs) transfected with Snai1 small interfering RNA (siRNA) or negative control (NC) siRNA, as determined by RNA sequencing. **b** Schematic illustration of collagen crosslinking. **c–f** Protein levels of collagen IV and the crosslinking enzyme Lox in PCs after Snai1 knockdown or overexpression (*n* = 3). **g** Chromatin immunoprecipitation (ChIP)-quantitative real-time (qRT)-PCR showing Snai1 binding to the promoters of Col2a1, Col4a1 and Lox in PCs, and the enrichment was quantified (*n *= 3). Neg represents negative control regions. **h** Proliferation of bEnd.3 cells co-cultured with PCs overexpressing Snai1 and with Col4a1 knockdown, determined by immunofluorescence staining for Pecam1 and EdU. **i** Quantification of proliferating bEnd.3 cells (*n* = 3). **j** Tube formation of bEnd.3 cells co-cultured with PCs as in **h**. **k** Quantification of loops and junctions in **j** (*n* = 3). **l** Proliferation of bEnd.3 cells co-cultured with PCs overexpressing Snai1 and with β-aminopropionitrile (BAPN) treatment, determined by immunofluorescence staining for Pecam1 and EdU. **m** Quantification of EdU^+^ bEnd.3 cells in **l** (*n* = 3). **n** Tube formation of bEnd.3 cells co-cultured with PCs treated as in **l**. **o** Quantification of loops and junctions in **n** (*n* = 3). Data are presented as mean ± SD. Statistical significance was determined by two-tailed Student’s *t* test (**e**, **f**) and one-way analysis of variance followed by Tukey’s post hoc test (**g**, **i**, **k**, **m** and **o**). *P  <  0.05; **P  <  0.01; ***P  <  0.001; ****P < 0.0001; ns not significant.

To define the role of Snai1 in ECM remodelling, we performed RNA sequencing on pericytes following Snai1 knockdown. The expression of multiple collagen genes (e.g. Col4a1, Col6a3) and key collagen crosslinking enzymes (Lox, Plod) was markedly downregulated upon Snai1 depletion (Fig. 5a). Collagen crosslinking, which is essential for ECM stability, requires both collagen monomers as substrate and enzymes such as Lox/Plod as catalysts^8,9^ (Fig. 5b). Consistent with the sequencing data, qRT-PCR and western blotting confirmed that Snai1 knockdown reduced the mRNA and protein levels of specific collagens (Col4a1, Col2a1) and crosslinking enzymes (Lox, Plod) in pericytes (Fig. 5c–f and Supplementary Fig. 6f). As Snai1 is a transcription factor and its putative binding motifs were identified in promoters of key ECM genes (Supplementary Fig. 6g), we performed chromatin immunoprecipitation assays, which confirmed direct binding of Snai1 to the promoter regions of key ECM genes, including Col2a1, Col4a1 and Lox (Fig. 5g). Furthermore, knockdown of Col4a1 or pharmacological inhibition of Lox activity with BAPN reversed the pro-angiogenic effects of Snai1-overexpressing pericytes on endothelial cells (Fig. 5h–o). In summary, Snai1 upregulation in pericytes drives a pro-angiogenic ECM signature by promoting collagen synthesis and crosslinking.

### Snai1 upregulation in pericytes drives pathological extracellular matrix deposition and collagen crosslinking in vivo

**Fig. 6:**
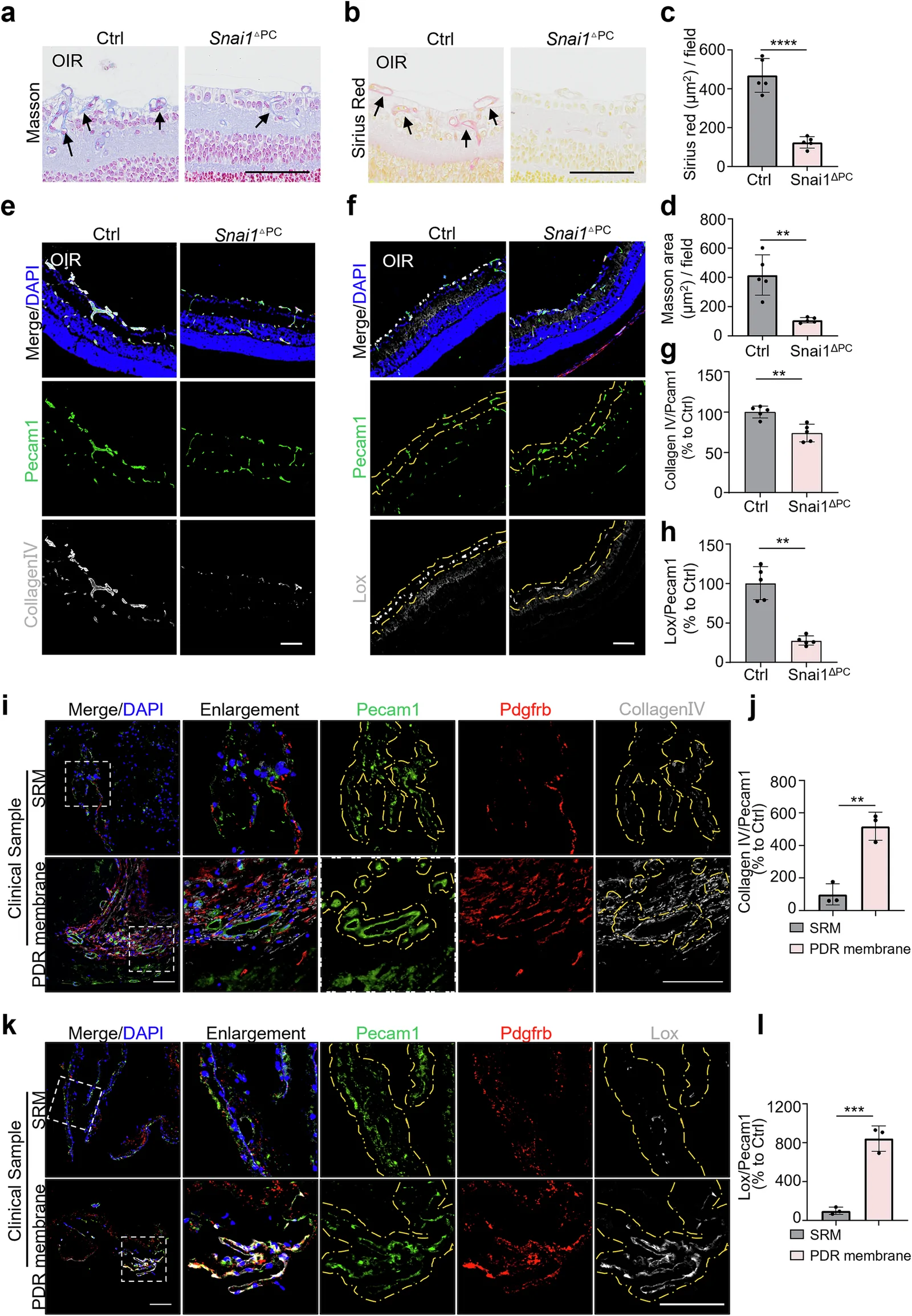
Snai1 deficiency in pericytes attenuates collagen deposition and crosslinking in oxygen-induced retinopathy. **a**, **b** Masson’s trichrome and Sirius red staining of retinal sections in control (Ctrl) and *Snai1*^△PC^ oxygen-induced retinopathy (OIR) mice. **c, d** Quantification of collagen deposition in **a** and **b**, respectively (*n* = 5). **e** Immunofluorescence staining for Pecam1 and Collagen IV in retinal sections. **f** Immunofluorescence staining for Pecam1 and Lox in retinal sections. Perivascular regions (0–20 μm around Pecam1⁺ vessels) are outlined by yellow lines for quantification. **g**, **h** Ratio of Collagen IV^+^ or Lox^+^ areas to Pecam1^+^ areas compared with the Ctrl group (*n* = 5). **i** Immunofluorescence staining of subretinal membrane (SRM) and proliferative diabetic retinopathy (PDR) membrane for Pecam1, Pdgfrb, Collagen IV and DAPI. **j** Ratio of Collagen IV^+^ areas to Pecam1^+^ areas (*n* = 3). **k** Immunofluorescence staining of SRM and PDR membrane for Pecam1, Pdgfrb and Lox. **l** Ratio of Lox^+^ areas to Pecam1^+^ areas (*n* = 3). Yellow lines demarcate the perivascular regions (0–20 μm surrounding Pecam1^+^ vessels) for quantification. Scale bar: 100 μm (**a**, **b**, **e**, **f**, **i** and **k**). Data are presented as mean ± SD. Statistical significance was determined by two-tailed Student’s *t* test (**c**, **d**, **g**, **h**, **j** and **l**). **P  <  0.01; ***P < 0.001; ****P  <  0.0001.

We next assessed whether upregulated Snai1 regulates collagen synthesis and crosslinking in the OIR model in vivo. Masson’s trichrome and Picrosirius staining revealed a significant reduction in total collagen deposition in the retinas of *Snai1*^ΔPC^ OIR mice compared with the controls (Fig. 6a–d). Immunofluorescence of retinal sections showed that Snai1 knockout also reduced the perivascular enrichment of type IV collagen, the major basement-membrane (BM) component, and decreased the level of the crosslinking enzyme Lox (Fig. 6e–h). In whole-mounted retinas from the control OIR mice, type IV collagen formed dilated, thickened sleeves around neovascular tufts. In contrast, *Snai1*^ΔPC^ OIR retinas exhibited a more normalized, continuous pattern of type IV collagen (Supplementary Fig. 6h). Transmission electron microscopy further confirmed a thinner and more organized BM structure in the knockout group (Supplementary Fig. 6i, j).

Consistently, analysis of human clinical samples revealed elevated levels of Col4a1 and Lox in the vitreous liquid from patients with diabetic retinopathy compared with controls (Supplementary Fig. 7a, b). Immunofluorescence further confirmed higher expression of type IV collagen and Lox in diabetic retinopathy proliferative membranes versus non-neovascular subretinal membranes (Fig. 6i–l), supporting collagen deposition and crosslinking as a hallmark of RNV. Collectively, these data demonstrate that Snai1 upregulation in pericytes promotes excessive collagen deposition and crosslinking, which disrupts vascular BM architecture during pathological RNV in vivo.

### Hypoxic endothelial cells upregulate pericyte Snai1 via ANGPT2/TGFβ1-mediated protein stabilization

**Fig. 7:**
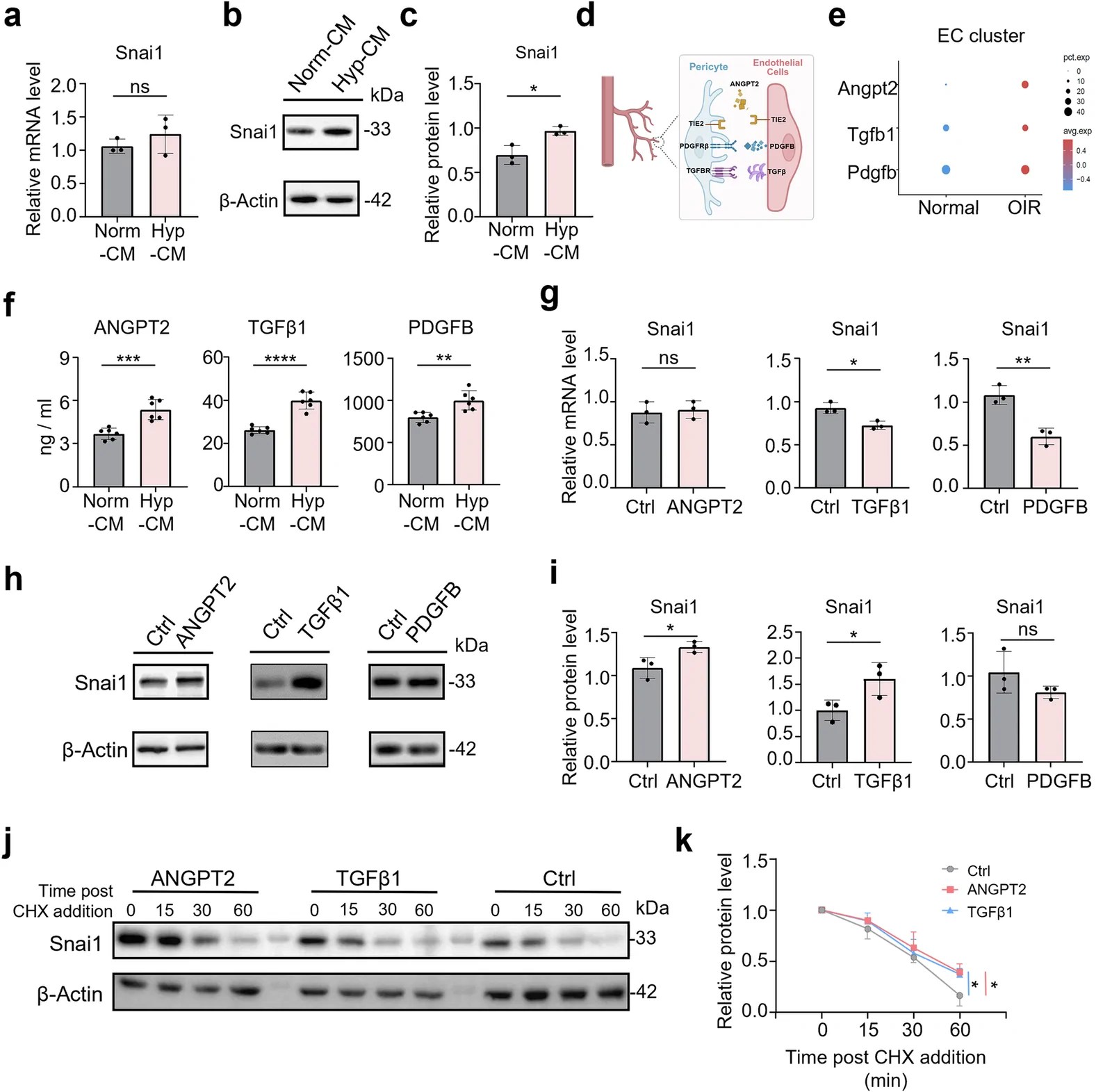
Hypoxic endothelial cells upregulate Snai1 expression in pericytes via angiocrine factors. **a–c** mRNA and protein levels of Snai1 in pericytes (PCs) treated with normoxic or hypoxic endothelial cell (EC)-conditioned medium (CM) (*n* = 3). **d** Schematic illustration of EC–PC crosstalk that may induce Snai1 in PCs. **e** Expression of selected angiocrine factors in normal and oxygen-induced retinopathy (OIR) ECs, based on single-cell RNA sequencing data from normal and OIR retinas (BioProject: PRJNA864092). **f** ELISA analysis of ANGPT2, TGFβ1 and PDGFB levels in normoxic versus hypoxic EC-conditioned medium (*n* = 6). **g–i** Snai1 mRNA and protein expression in PCs treated with ANGPT2, TGFβ1 and PDGFB (*n* = 3). **j**, **k** Snai1 protein stability assessed by cycloheximide (CHX) treatment after ANGPT2 and TGFβ1 stimulation (*n* = 3). Data are presented as mean ± SD. Statistical significance was determined by two-tailed Student’s *t* test (**a**, **c**, **f**, **g**, **i** and **k**). *P  <  0.05; **P  <  0.01; ***P  <  0.001; ****P  <  0.0001; ns not significant.

Given the pro-angiogenic role of pericyte Snai1, we investigated its upstream regulation. RNV involves dual signalling between pericytes and endothelial cells. We therefore hypothesized that endothelial cell-derived signals under hypoxia might induce Snai1 in pericytes. Treating pericytes with conditioned medium from hypoxic endothelial cells did indeed significantly increase the Snai1 protein level, but did not markedly affect its mRNA level (Fig. 7a–c). Furthermore, we focused on three candidate endothelial cell-secreted factors known to influence pericytes: ANGPT2, TGFβ1 and PDGFB^23^ (Fig. 7d). Transcriptomic data confirmed their upregulation in OIR endothelial cells, and ELISA verified increased secretion under hypoxia (Fig. 7e, f). Treating pericytes with each factor showed that only ANGPT2 and TGFβ1 increased Snai1 protein level, without affecting its mRNA level (Fig. 7g, i). Previous studies have shown that ANGPT2 and TGFβ1 can promote the expression of downstream target genes by suppressing their degradation^24–26^. To determine whether the observed upregulation resulted from reduced degradation, we treated cells with CHX to block de novo protein synthesis, in the presence of ANGPT2 or TGFβ1 stimulation, and examined the decay kinetics of the target protein. The degradation of Snai1 protein was slower in treated groups, indicating that both factors primarily stabilize Snai1 protein by inhibiting its degradation rather than promoting its transcription (Fig. 7j, k). We next examined the effects of ANGPT2 and TGFβ1 on the expression of Col4a1 and Lox. Consistent with the results of Snai1 overexpression, both factors significantly upregulated these genes (Supplementary Fig. 8a–d).

To further investigate the receptor mediating the effect of ANGPT2 on pericytes, we compared the expression of Tie2 and integrin subunits between endothelial cells and pericytes^27–30^. Pericytes exhibited lower Tie2, Itgav and Itgb3 levels, higher Itgb1 level, and comparable Itga5 expression relative to endothelial cells (Supplementary Fig. 8e). Importantly, blocking integrin α5β1 with ATN161 abolished ANGPT2-induced Snai1 upregulation (Supplementary Fig. 8f), indicating that integrin α5β1 may mediate ANGPT2 signalling in pericytes.

In summary, these results identify ANGPT2 and TGFβ1, secreted by hypoxic endothelial cells, as key upstream signals that post-transcriptionally stabilize Snai1 protein in pericytes.

### Targeting collagen crosslinking attenuates pathological neovascularization and restores retinal function

**Fig. 8:**
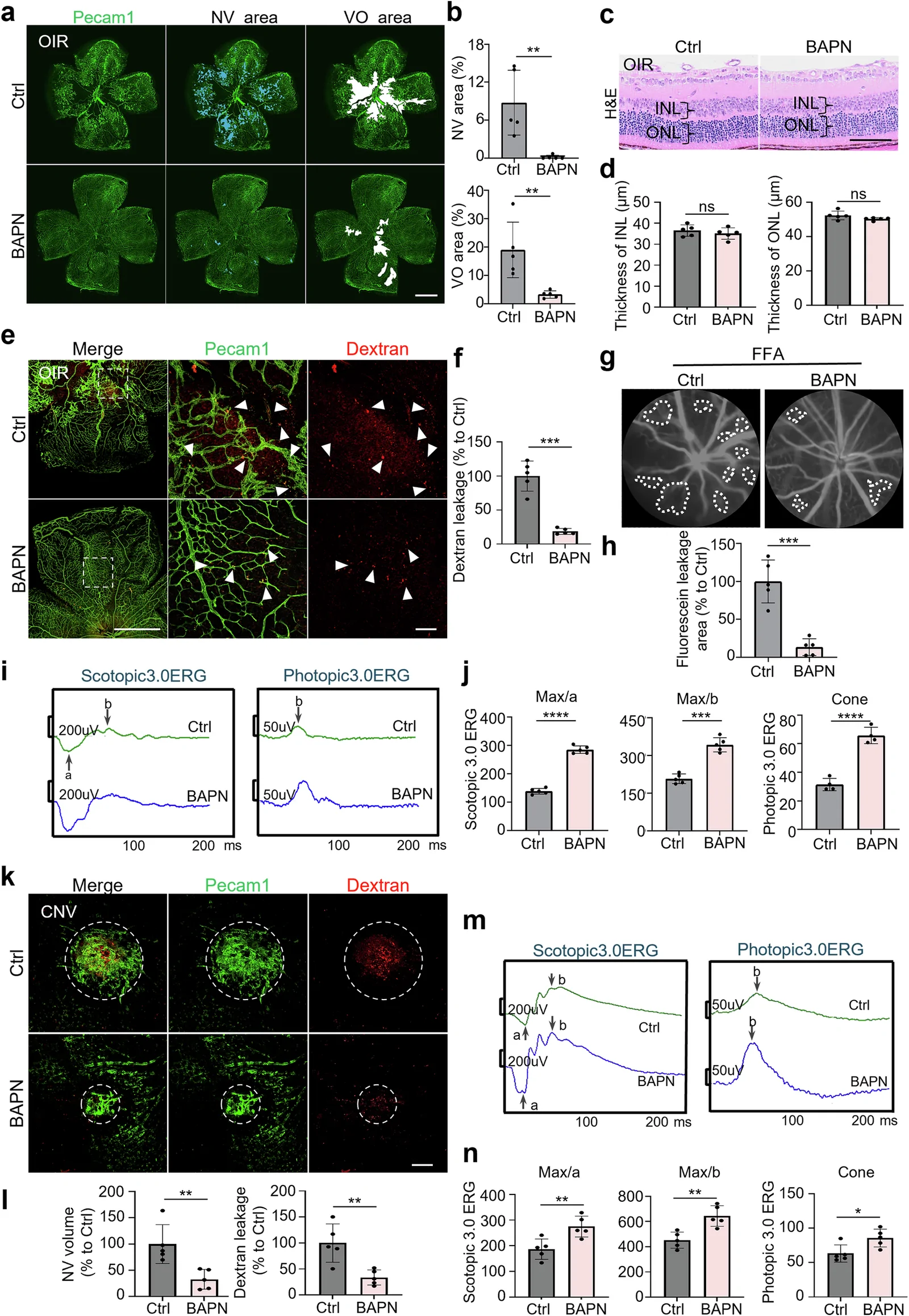
Targeting collagen crosslinking suppresses pathological angiogenesis in vivo. **a** Retinal flat-mounts stained for Pecam1 in phosphate buffered saline (PBS)- and β-aminopropionitrile (BAPN)-injected oxygen-induced retinopathy (OIR) mice. Blue: neovascular (NV) tufts; white: vaso-obliteration (VO) area. **b** Quantification of NV and VO area (*n* = 5). **c** Haematoxylin and eosin (H&E) staining of retinal sections from PBS- and BAPN- injected OIR mice. **d** Quantitative analysis of the inner nuclear layer (INL) and outer nuclear layer (ONL) thickness (*n* = 5). **e** Pecam1 and dextran staining of retinal flat-mounts (PBS versus BAPN). Arrowheads indicate dextran leakage. **f** Quantification of leakage areas, normalized to control (Ctrl) (*n* = 5). **g** Fundus fluorescein angiography (FFA) showing capillary leakage (dashed lines) treated with PBS or BAPN. **h** Capillary leakage areas were quantified and normalized to Ctrl (*n* = 5). **i** Electroretinography waveforms from the PBS- and BAPN-treated OIR mice. **j** Quantification of amplitudes of waves indicated in **i** (*n* = 5). **k** Choroidal flat-mounts stained for Pecam1 and dextran in PBS- and BAPN-treated CNV mice. **l** Quantification of NV volume and dextran leakage (*n* = 5). **m** Electroretinography waveforms from the PBS- and BAPN-treated CNV mice. **n** Quantification of amplitudes of waves indicated in **m** (*n* = 5). Scale bar: 1 cm (**a** and overview of **e**) and 100 μm (**c**, enlarged **e** and **k**). Data are presented as mean ± SD. Statistical significance was determined by two-tailed Student’s *t* test (**b**, **d**, **f**, **h**, **j**, **l** and **n**). *P  <  0.05; **P  <  0.01; ***P  <  0.001; ****P  <  0.0001; ns not significant.

Given the observed increase in pericyte-derived abnormal collagen deposition and crosslinking during pathological RNV, we investigated its potential as a therapeutic target. In the OIR model, intravitreal injection of BAPN at P12 not only significantly reduced neovascular and vaso-obliterated areas and promoted vascular normalization but also markedly diminished pathological neovascular tufts, with no apparent retinal toxicity (Fig. 8a–d). Moreover, BAPN treatment yielded functional improvements, as demonstrated by reduced vascular leakage and enhanced retinal responses on electroretinography (Fig. 8e–j). We further extended our evaluation to a laser-induced CNV mouse model, in which BAPN consistently demonstrated therapeutic efficacy, as reflected by reduced CNV lesion volumes, diminished pathological vascular leakage and improved neural retinal function (Fig. 8k–n). Altogether, these findings demonstrate that targeting the collagen crosslinking enzyme effectively inhibits pathological angiogenesis and promotes functional recovery, highlighting its therapeutic promise for pathological ocular neovascularization.

## Discussion

This study elucidates a novel and significant role of crosstalk between pericytes and endothelial cells in pathological neovascularization during RNV. We show for the first time that Snai1 upregulation drives pericyte reprogramming from a quiescent state maintaining vascular stability into an activated state promoting angiogenesis via remodelling the ECM in RNV. Mechanistically, Snai1 promotes the transcription of collagens and crosslinking enzyme Lox, leading to excessive deposition and abnormal crosslinking of the ECM. This aberrant ECM remodelling establishes a pro-angiogenic microenvironment and deteriorates the contact-dependent physiological interactions between endothelial cells and pericytes, and consequently, driving disorganized neovascularization (Supplementary Fig. 9). Importantly, either specifically knockout Snai1 in pericytes or pharmacological inhibiting collagen crosslinking significantly suppresses pathological neovascularization and promotes functional vascular remodelling, providing a novel strategy for “vascular normalization” therapy for RNV-related diseases.

### Pericytes play an essential role in retinal neovascularization

Pericytes are pivotal regulators of vascular integrity and blood flow. Emerging evidence has elucidated that pericytes profoundly contribute to RVDs by vascular detachment, hypercontraction and myofibroblast differentiation^5,31,32^. The present study identified dysfunctional pericytes in RNV using the OIR model, in which pericytes lost coverage to vessels and exhibited an enlarged cell body, accompanied by profound functional reprogramming. Advances in single-cell approaches facilitate identification of distinct molecular profiles in these pericytes, including rearranged cytoskeleton, modified focal adhesion, enhanced protein translation, and activation of the ECM and Hippo signalling. Reorganization of cytoskeleton and focal adhesion compromises adhesion between pericytes and endothelial cells, probably leading to pericyte detachment. Pericyte coverage followed by BM deposition is pivotal for vessel maturation^33^. Reduced coverage destabilizes the vasculature and increases the risk of its collapse, regression and fibrosis^34,35^. Activation of the Hippo pathway is known to strengthen the proliferation and survival of pericytes^36^. Enlarged cell bodies reflect an activated state characterized by elevated protein translation, which supports large-scale synthesis of secretory proteins such as ECM components, consequently leading to establishment of a pro-angiogenic niche via ECM modification.

### Snai1 induces pericytes to transit from a vessel maintainer into an angiogenic promoter

Snai1 is a well-known mediator of epithelial–mesenchymal transition (EMT). We have previously reported that Snai1 in endothelial cells facilitates the early stage of angiogenesis by enhancing endothelial invasiveness and migration^11^. Additionally, studies have elucidated that Snai2, another member of the Snail family, could activate pericytes and promote glioma progression^37^. We initially hypothesized that Snai1 induced analogous pericyte–myofibroblast transition (PMT), a process implicated in capillary leakage and retinal fibrosis^38^. Surprisingly, overexpressing Snai1 exerted a slight influence on pericyte proliferation, migration or myofibroblast marker expression. Instead, Snai1 upregulation in pericytes enhanced the angiogenic ability of endothelial cells in a contact-dependent manner. Although previous studies indicated that pericyte loss facilitated endothelial-cell sprouting and disorganized neovascularization^39^, our present work revealed a novel role of pericyte–endothelial-cell contact during angiogenesis. In the early phase, pericytes appear to release “contact inhibition”, initiating endothelial-cell sprouting and proliferation. Although in the following stages, recruited pericytes provide structural and biochemical support for endothelial cells via contact interaction, endothelial-cell-derived signalling, termed angiocrine signalling, is well known to instruct pericyte behaviour. In our in vitro model, pericytes exerted a relatively modest effect on endothelial-cell tube formation compared with the robust in vivo phenotype. This discrepancy probably reflects: (1) the greater efficacy of complete genetic knockout in vivo than transient siRNA-mediated knockdown in vitro; and (2) the enhanced complexity of the in vivo environment, which includes additional cellular players absent from the two-cell co-culture system^40,41^.

We further observed that pericyte-specific Snai1 knockout did not impair physiological angiogenesis. We interpret this as a consequence of homeostatic compensation — multiple cell types jointly maintain a balanced extracellular milieu that buffers Snai1 loss under physiological conditions. Under pathological stress, however, this compensatory capacity is overwhelmed, revealing Snai1’s essential role in pathological NV^34^. Our simplified in vitro system, devoid of such buffering networks, captures a direct yet simplified effect. Additionally, although we detected no significant changes in pericyte proliferation, migration or transdifferentiation following Snai1 loss, we acknowledge that cell-autonomous effects in other dimensions cannot be ruled out. Given the high plasticity of pericytes, future multi-omics approaches may identify additional Snai1-regulated functional modalities^5,20^.

Our study demonstrated that hypoxic endothelial cells upregulate Snai1 in pericytes via paracrine. Dysregulation of typical endothelial-cell–pericyte crosstalk pathways — such as Angpt1/2-Tie2, Tgfβ and Pdgfb/Pdgfrβ — has been well established^23^. Surprisingly, we found that ANGPT2 and TGFβ1 can probably regulate Snai1 by inhibiting its degradation, although the detailed molecular mechanism remains to be investigated^42,43^. In summary, the present study uncovers a self-amplifying cycle that sustains pathological retinal angiogenesis. Hypoxia endothelial cells upregulate Snai1 in pericytes through paracrine signalling, whereas Snai1 upregulation triggers pericytes toward a pro-angiogenic phenotype, thereby perpetuating the angiogenic endothelial-cell activation. Previous studies indicate that ANGPT2 can signal via both Tie2 and integrin receptors, including αvβ3 and α5β1 (refs.^27–30^). Our findings indicated that ANGPT2 enhances Snai1 expression in pericytes specifically through integrin α5β1. Regarding TGFβ1, it is well established that this cytokine signals through the TβRI/TβRII receptor complex, leading to Snai1 upregulation via both Smad-dependent and Smad-independent routes^44^. Although our data have identified these receptor-level mechanisms, we acknowledge that the detailed signalling cascade downstream of ANGPT2 and TGFβ1 in pericytes remains to be fully elucidated and needs further exploration.

### Abnormal extracellular matrix scaffold, formed by excessive collagen deposition and crosslinking, mediates the pro-angiogenic effects of pericytes

Our work revealed that knockout Snai1 in pericytes effectively restores vascular function and ECM structure, as demonstrated by reduced vascular leakage, improved perfusion and decreased excessive crosslinking. Collagen crosslinking has emerged as a crucial regulator of angiogenesis. Specifically, it provides mechanical cues for endothelial cells, guiding them to directed migration, proliferation and sprouting through the establishment of stiffness gradients, promotion of VEGF release, upregulation of metalloproteases activity and disruption of tight junctions^45–47^. The present study found that collagen crosslinking can also mediate the angiogenic activity of pericytes. Crosslinking fostered by pericytes remodels the ECM, thereby modulating ECM–receptor signal transduction^48^. For example, a mimetic derivative of type IV collagen (AXT107) can downregulate phosphorylation of VEGFR2, c-Met and PDGFRβ, thereby enhancing vascular stability^49^. Our study suggested that collagen crosslinking influences angiogenesis through a multicellular mechanism, involving endothelial cells and pericytes, which provides a multidimensional perspective on how ECM regulates pathological neovascularization. Of note, the in vivo expression profiles of different collagens appear more intricate than those observed in vitro, as exemplified by the opposing trends of Col2a1 and Col4a1 in the vitreous humour of patients with diabetic retinopathy (Supplementary Fig. 7b). This apparent discrepancy can be attributed to their distinct cellular sources and regulatory mechanisms^50–52^. These divergent trends probably reflect the tight and independent regulation of different collagens within the in vivo milieu. Further investigations are needed to clarify the precise expression dynamics of various collagens in the human vitreous throughout diabetic retinopathy progression.

### Inhibiting collagen crosslinking to promote vascular normalization holds great therapeutic value for retinal neovascularization

Currently, “inhibiting neovascularization” is the mainstream therapeutic strategy for RNV. However, excessive pruning of vessels would lead to tissue hypoxia, which significantly compromises the treatment outcome^53^. In this context, Rakesh K. Jain proposed the concept of “vascular normalization”, which is aimed not only at inhibiting pathological angiogenesis but also at restoring vascular structural and functional homeostasis^54,55^. Accumulating evidence has demonstrated that manipulating vascular normalization could augment therapeutic efficacy by improving delivery of anti-tumour drugs and immune cells^56,57^. Additional benefits include modulation of the immunosuppressive microenvironment, enhancement of combination therapies and reduction of systemic toxicity^58^. Achieving vascular normalization requires a delicate balance between pro- and anti-angiogenic factors, with the timing and dosage of anti-angiogenic agents posing a central clinical challenge. Here, we revealed that targeting the pericyte Snai1–ECM remodelling axis significantly facilitates vascular normalization. This approach reconstructs the vasculature towards a more physiological state rather than merely inhibiting neovascularization, which further enhances perfusion and supports neural recovery. Besides, our research offers a novel therapeutic perspective for vascular normalization: restoring the vascular microenvironment through ECM remodelling.

### Limitations and future perspectives

Although the OIR model is widely used for investigating RNV, previous study has revealed that only a small similarity was addressed between the OIR model and the neovascular membranes of human PDR at transcription level^18^. Besides, we demonstrated that Snai1 disrupts endothelial-cell–pericyte crosstalk through ECM remodelling. Consequently, it would be important to further explore the downstream pathways that activate endothelial cells. Furthermore, developing a targeted delivery approach to pericytes represents a key translational challenge. In summary, our study highlights potential strategies such as targeting pericytes and remodelling ECM for the treatment of RNV.

## Supplementary information

Supplementary Information

## Data Availability

Raw RNA sequencing data have been deposited in the Sequence Read Archive database under accession number PRJNA1416461. The remaining data and materials related to this paper may be obtained from the authors upon reasonable request.
