# Supplementary material for: Pericyte Snai1 ablation prevents pathological angiogenesis in retinopathy via extracellular matrix remodelling-mediated inhibition of endothelial cell activation
Source: Exp Mol Med. 2026 Oct 26;58(10):3609–25. doi: 10.1038/s12276-026-01874-1 (PMC13737156; doi:10.1038/s12276-026-01874-1)
Supplement: Supplementary file 1 [file 12276_2026_1874_MOESM1_ESM.pdf]

Supplementary Information

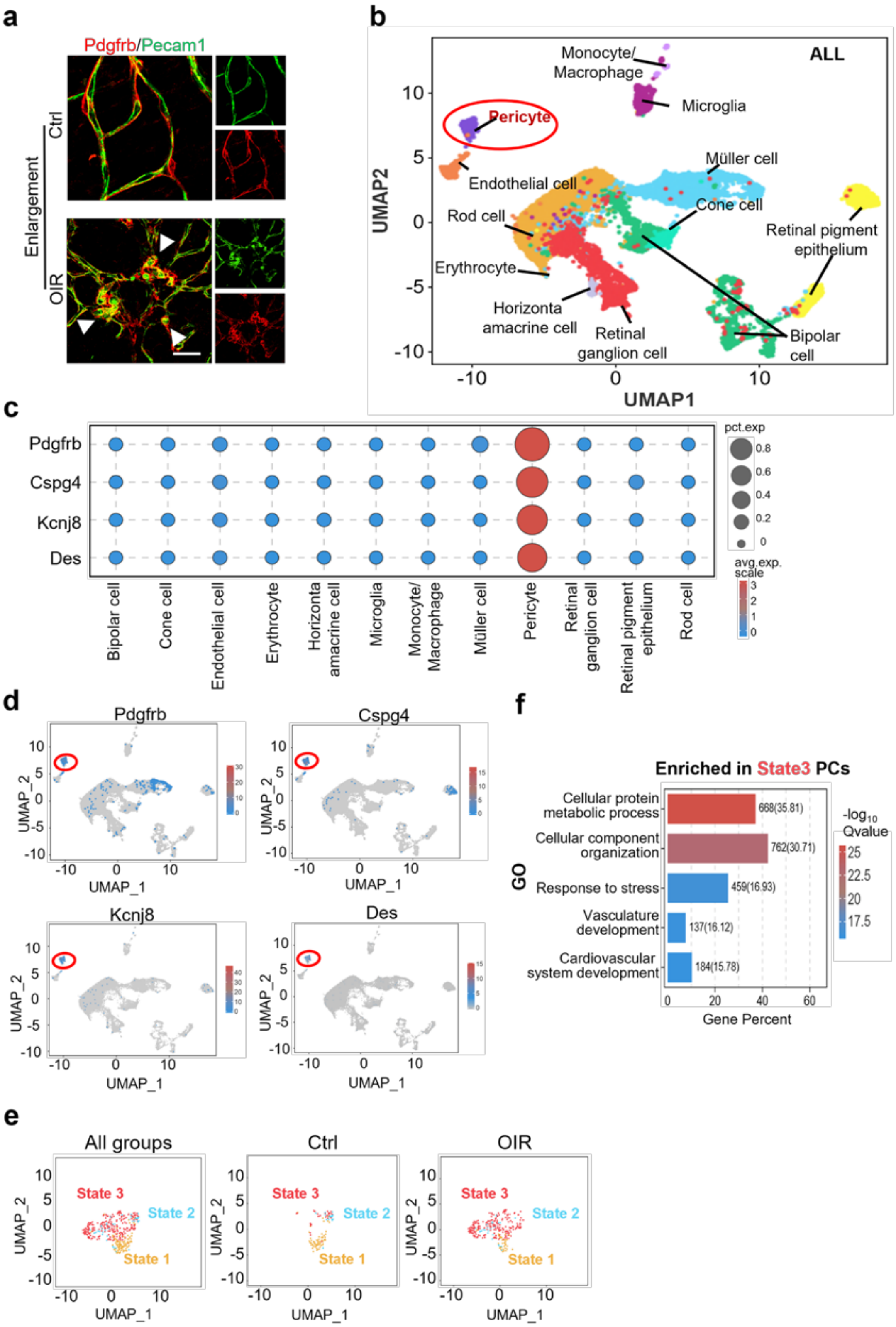

Supplementary Fig. 1. PCs transition from quiescent stabilizers to

pro-angiogenic activators in OIR.

(a) Enlarged immunofluorescence images of the retinal vasculature in control and OIR mice at P17. White arrowheads indicate PC aggregation. **(b)** UMAP projection of scRNA-seq data from normal and OIR mouse retinas at P17 (BioProject: PRJNA864092). **(c)** Bubble plot of canonical PC marker expression in retinal cell clusters. **(d)** Feature plots illustrating expression of PC markers across retinal cells. **(e)** UMAP projections showing PC subclusters (states 1-3) under Merged (All groups), Ctrl, and OIR datasets. **(f)** GO enrichment analysis of molecular function-related genes upregulated in State 3 PCs versus State 1 PCs. Scale bar: 10 μm.

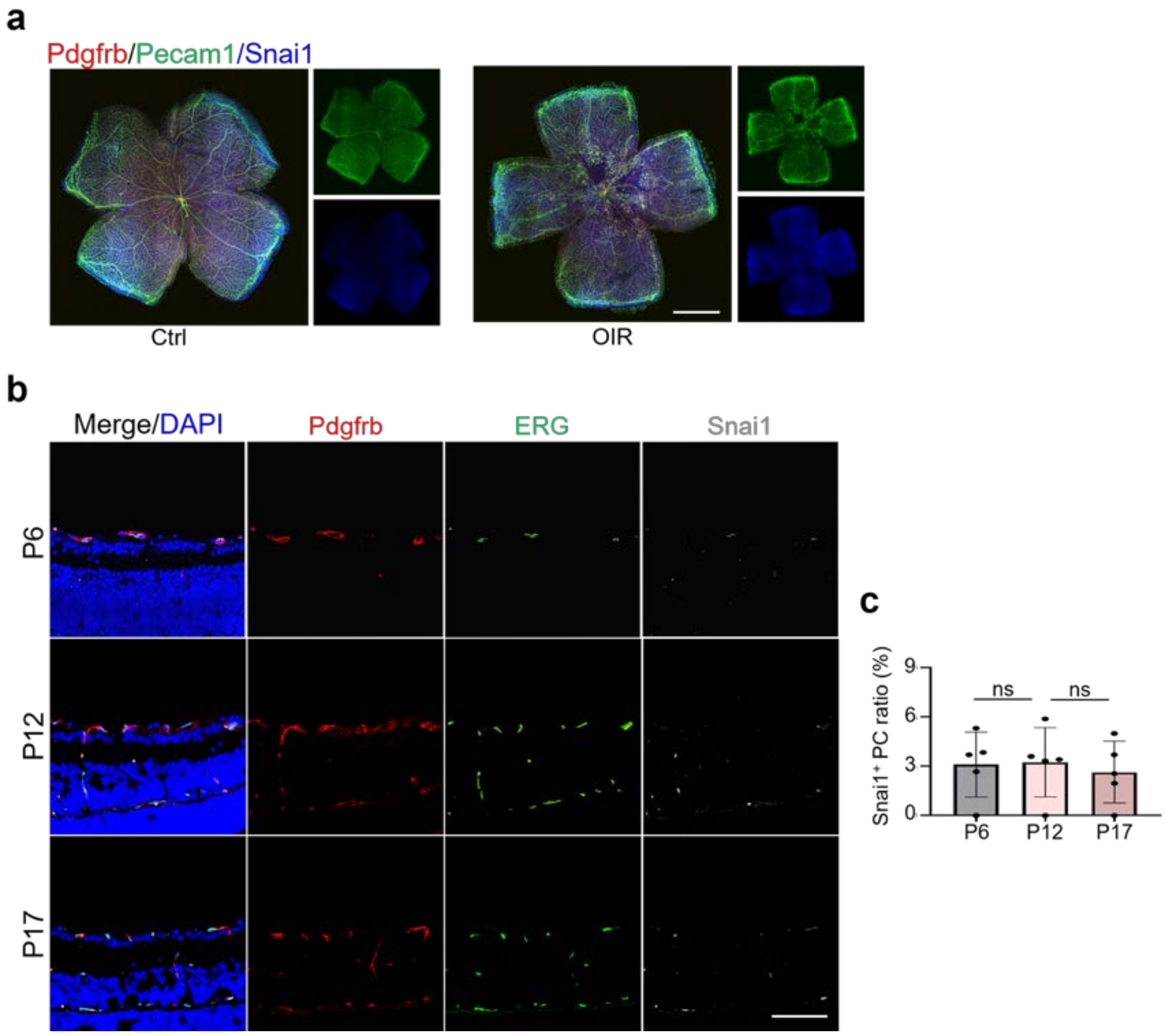

Supplementary Fig. 2. Snai1 expression is low in PCs during physiological retinal vascular development.

(a) Immunofluorescence of retinal flat-mounts from control and OIR mice at P17 stained for Pdgfrb, Pecam1 and Snai1. **(b)** Immunofluorescence of retinal cryosections from P6, P12, and P17 mice stained for Pdgfrb, ERG and Snai1. **(c)** Quantification of Snai1$^+$ PCs across developmental time points (n = 5). Scale bar: 1 cm (a); 100 μm (b). Data are presented as mean ± SD. Statistical significance was determined by one-way ANOVA with Tukey's post hoc test for multiple comparisons (c). ns, not significant.

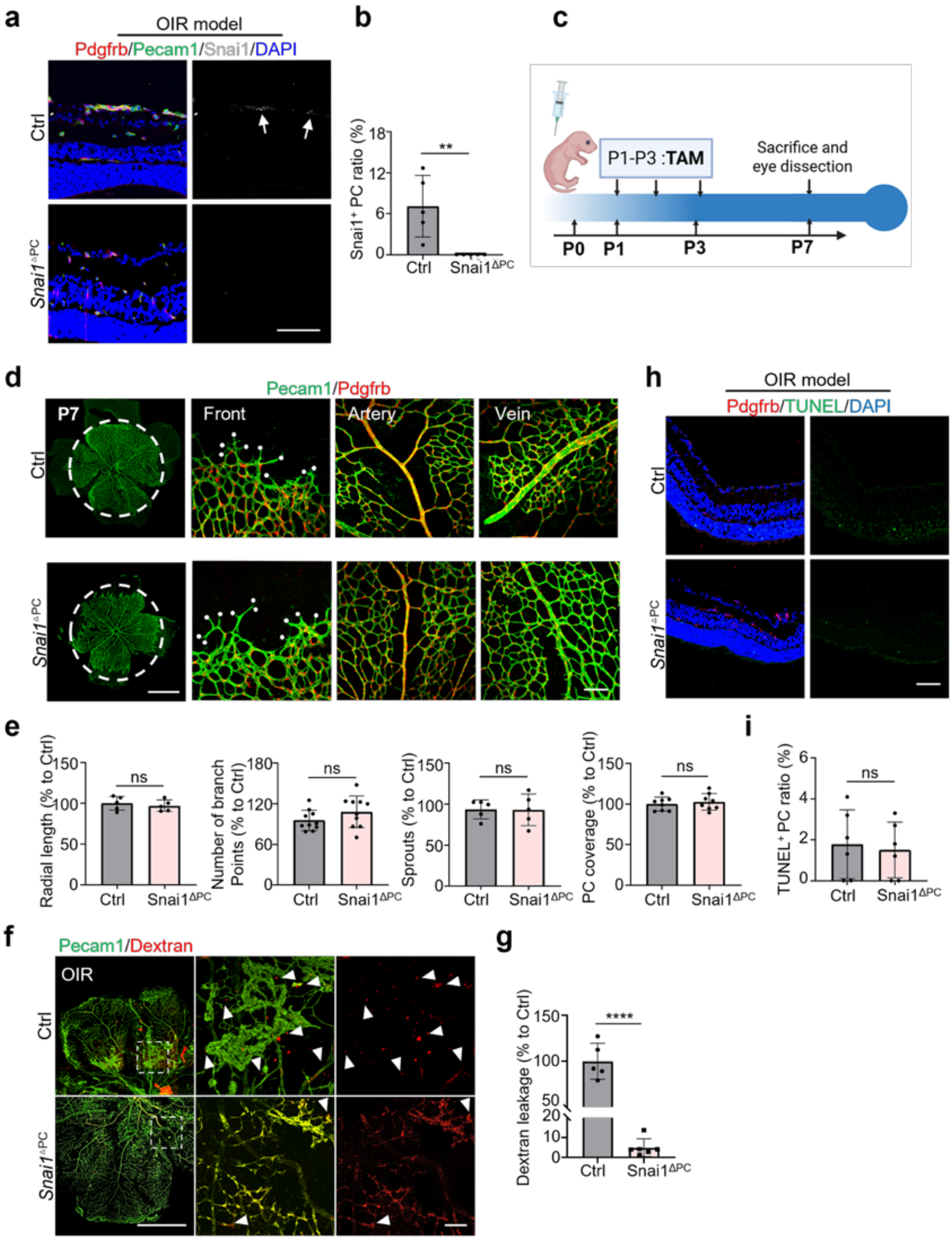

Supplementary Fig. 3. Snai1 knockout in PCs does not affect physiological retinal development or apoptosis.

(**a** and **b**) Immunofluorescence verifying Snai1 deletion in Pdgfrb$^+$ PCs in the OIR model, stained for Pdgfrb, Pecam1, Snai1, and DAPI. Arrows indicate Snai1

expression. (**c**) Schematic of the tamoxifen-induction pups (P1-P3) for assessing developmental angiogenesis at P7. (**d**) Whole-mount immunofluorescence for Pecam1 and Pdgfrb in control and *Snai1*$^{\triangle PC}$ retinas at P7. Images show higher magnification of vascular fronts, arteries, and veins. (**e**) Quantification of radial length, branch points, sprouts, and pericyte coverage (n = 5). (**f**) Pecam1 and Dextran staining of retinal flat-mounts in control and *Snai1*$^{\triangle PC}$ OIR mice. Arrowheads indicate Dextran leakage. (**g**) Quantification of leakage areas, normalized to control (n = 5). (**h**) Immunofluorescence for Pdgfrb, TUNEL, and DAPI in retinal sections. (**i**) Ratio of TUNEL$^+$ to Pdgfrb$^+$ cells (n = 5). Scale bar: 1 cm (overview of d and f) and 100 μm (a, magnified d, h, and magnified f). Data are presented as mean ± SEM. Statistical significance was determined by two-tailed Student's *t*-test (b, e, g and i). **, $p < 0.01$; ****, $p < 0.0001$; ns, not significant.

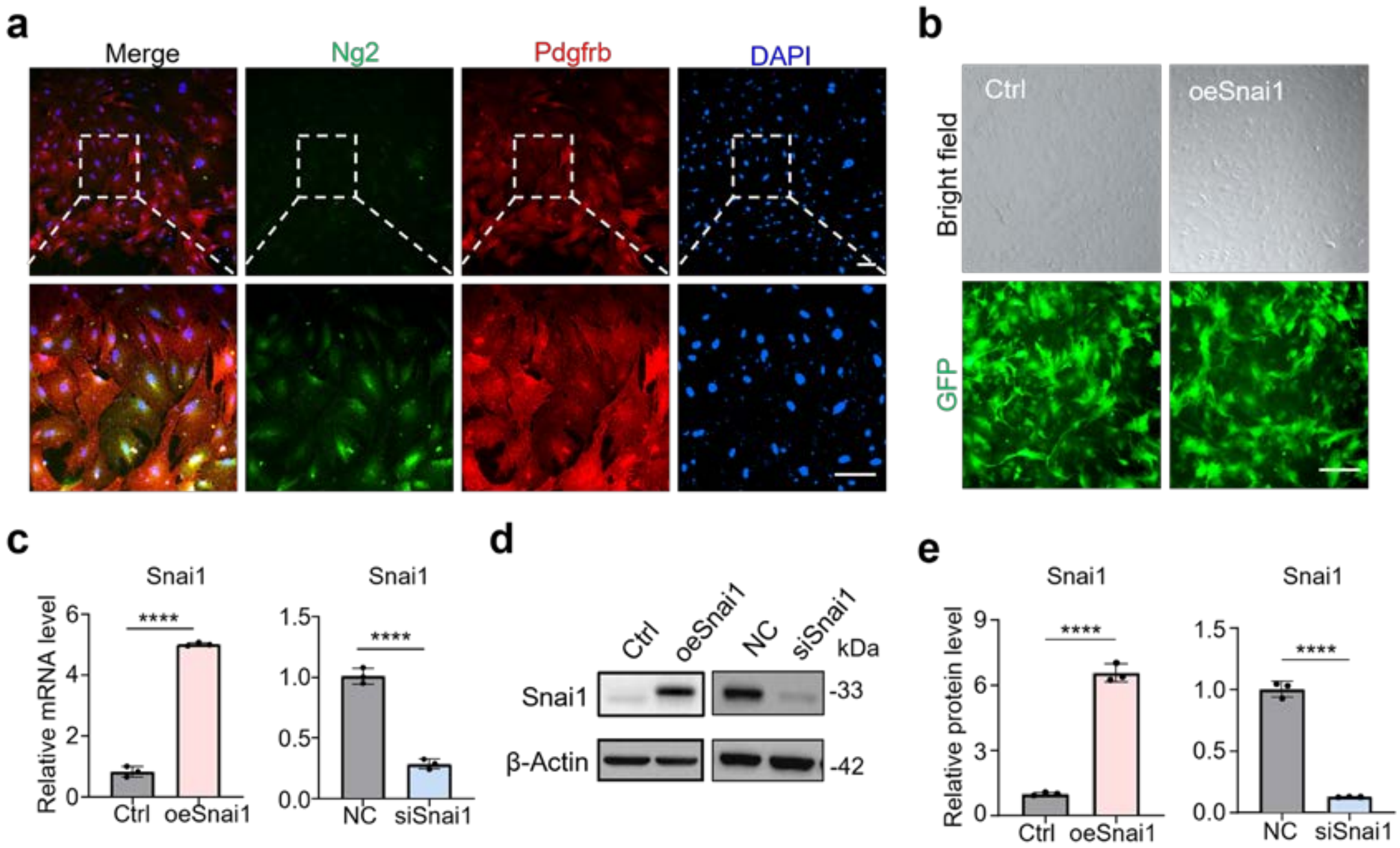

Supplementary Fig. 4. Primary PC identification and modulation of Snai1 expression.

(a) Immunofluorescence staining for canonical PC makers Ng2 and Pdgfrb. **(b)** Bright-field and fluorescence images confirming adenoviral infection efficiency (GFP signal). **(c-e)** mRNA and protein levels of Snai1 in PCs following Snai1 overexpression or siRNA knockdown (n = 3). Scale bar: 100 μm (a and b). Data are presented as mean ± SD. Statistical significance was determined by two-tailed Student's *t*-test (c and e). ****, $p < 0.0001$.

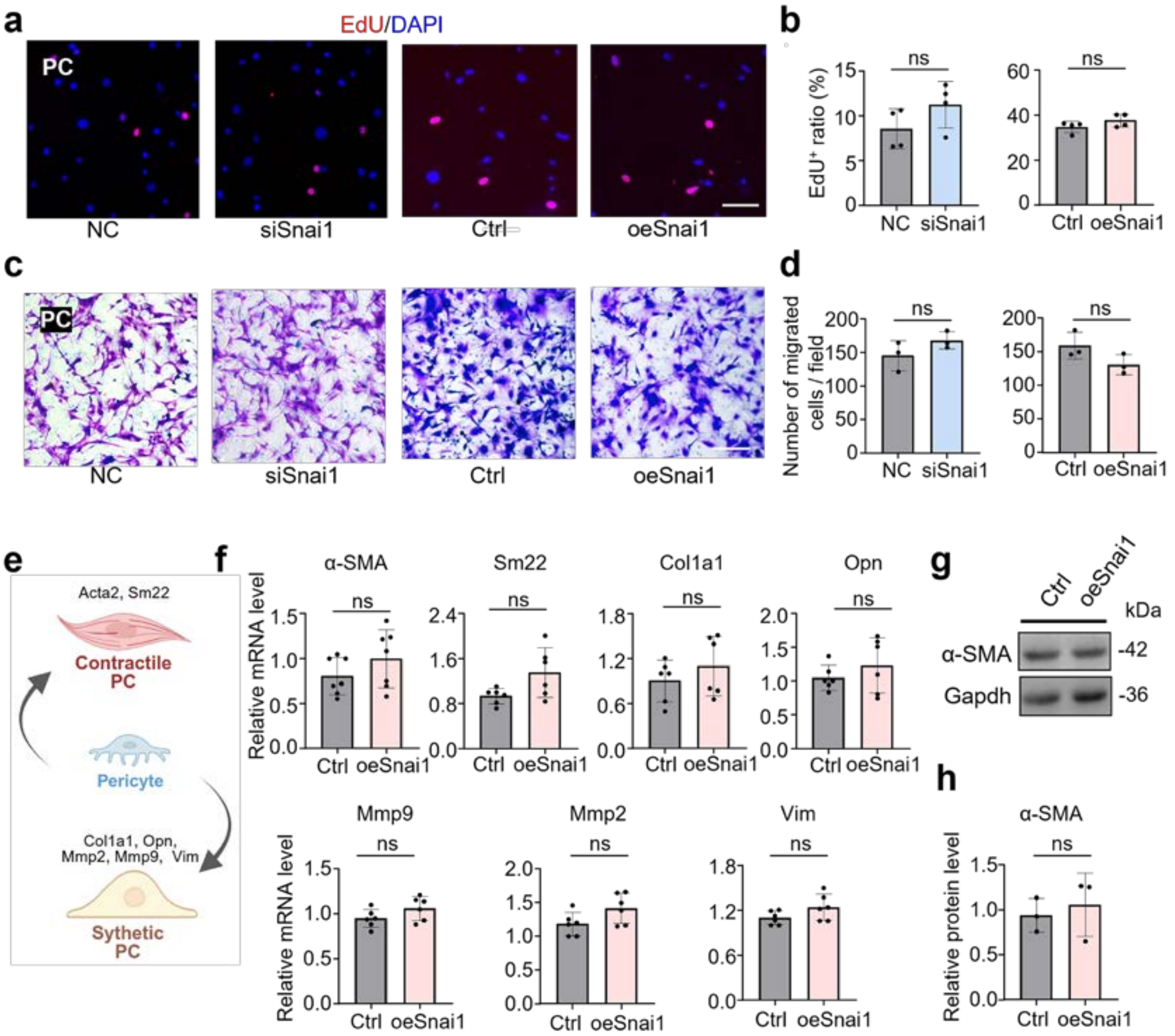

Supplementary Fig. 5. Snai1 has minimal effects on pericyte proliferation, migration, and phenotypic transition.

(a) EdU incorporation assays in PCs following Snai1 knockdown or overexpression. **(b)** Quantification of EdU^+ PCs (n = 4). **(c)** Transwell migration assay of PCs after Snai1 knockdown or overexpression. **(d)** Number of migrated PCs (n = 4). **(e)** Schematic of PC phenotypic states. **(f)** mRNA levels of contractile (Acta2, Sm22) and synthetic (Col1a1, Opn, Mmp9, Mmp2, Vim) markers in Snai1 overexpressing PCs (n = 6). **(g)** Protein expression of α-SMA in control and Snai1-overexpressing PCs. **(h)** Quantification of α-SMA protein level in (g) (n = 3). Scale bar: 100 μm (a and c). Data are presented as mean ± SD. Statistical significance was determined by two-tailed Student's *t*-test (b, d, f and h). ns, not significant.

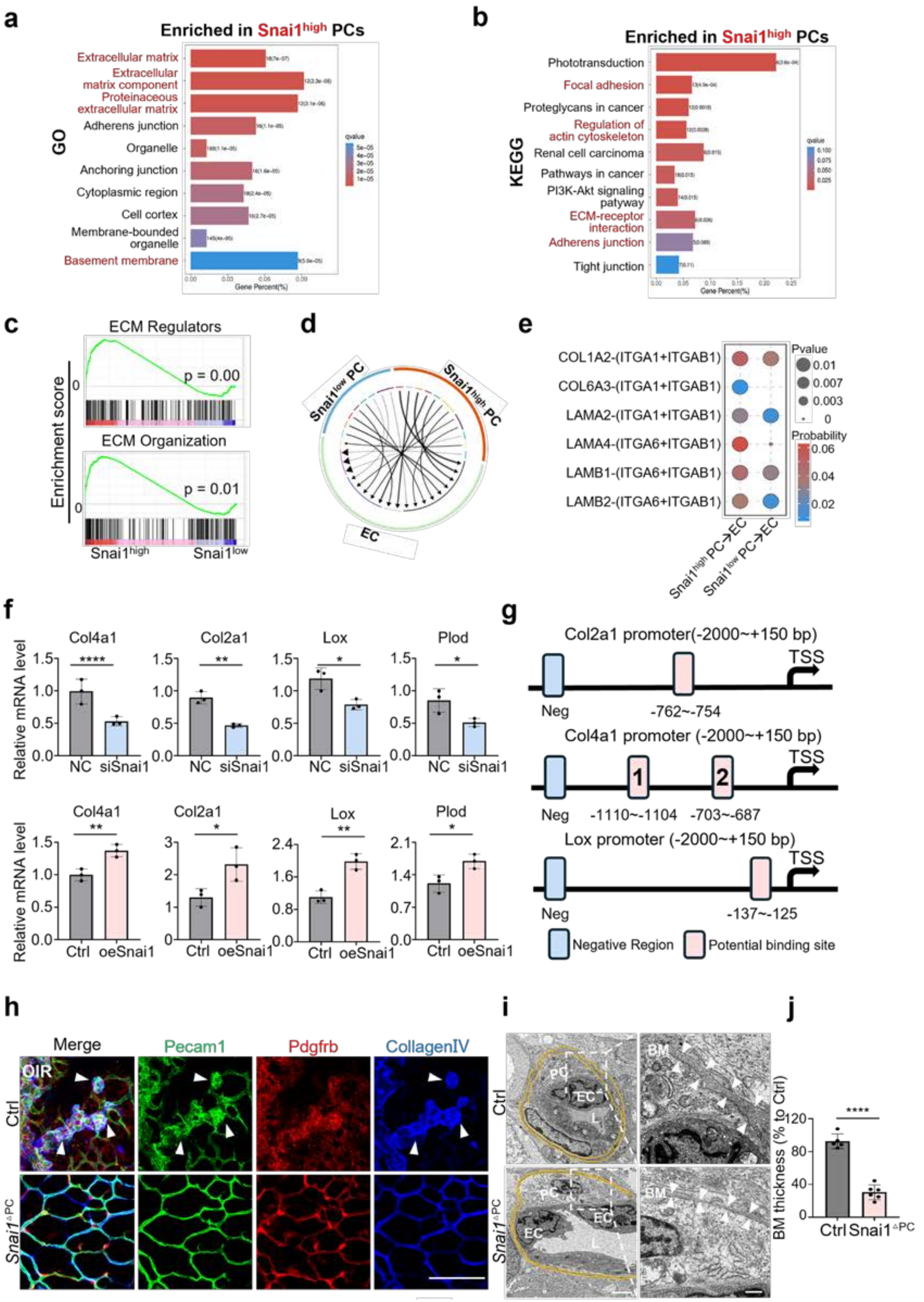

Supplementary Fig. 6. Snai1 in PCs regulates collagen and crosslinking enzyme expression to normalize ECM architecture.

(**a**) PCs were grouped by Snai1 expression (Snai1^{high} vs. Snai1^{low}). GO cell component

enrichment identified differentially expressed genes (DEGs) in Snai1^{high} PCs based on scRNA-seq data from normal and OIR retinas. **(b)** KEGG pathway enrichment of DEGs in Snai1^{high} PCs. **(c)** GSEA of ECM-related gene sets in Snai1^{high} and Snai1^{low} PCs. **(d)** Interaction network between Snai1^{high} or Snai1^{low} PCs and ECs. **(e)** Ligand-receptor interactions between Snai1^{high} or Snai1^{low} PCs and ECs. **(f)** mRNA level of collagen subunits (Col4a1, Col2a1) and crosslinking enzymes (Lox, Plod) of PCs with Snai1 overexpression or knockdown (n = 3). **(g)** Schematic of predicted Snai1-binding sites and negative control regions (Neg) in the promoters of Col2a1, Col4a1, and Lox. **(h)** Whole-mount immunofluorescence staining for Pecam1, Pdgfrb and DAPI in control and *Snai1*$^{\triangle PC}$ retinas. White arrows show thick, dilated type IV collagen sleeves. **(i)** TEM images of retinal section from control and *Snai1*$^{\triangle PC}$ OIR mice. Yellow lines delineate the basement membrane, and arrowheads indicate its thickness. **(j)** Quantification of BM thickness, normalized to control (n = 6). EC: endothelial cell; PC: pericyte; L: lumen; BM: basement membrane. Scale bar: 100 μm (h); 2 μm (left of i) and 0.5 μm (right of i). Data are presented as mean ± SD. Statistical significance was determined by two-tailed Student's *t*-test (f and j). *, $p < 0.05$; **, $p < 0.01$; ****, $p < 0.0001$.

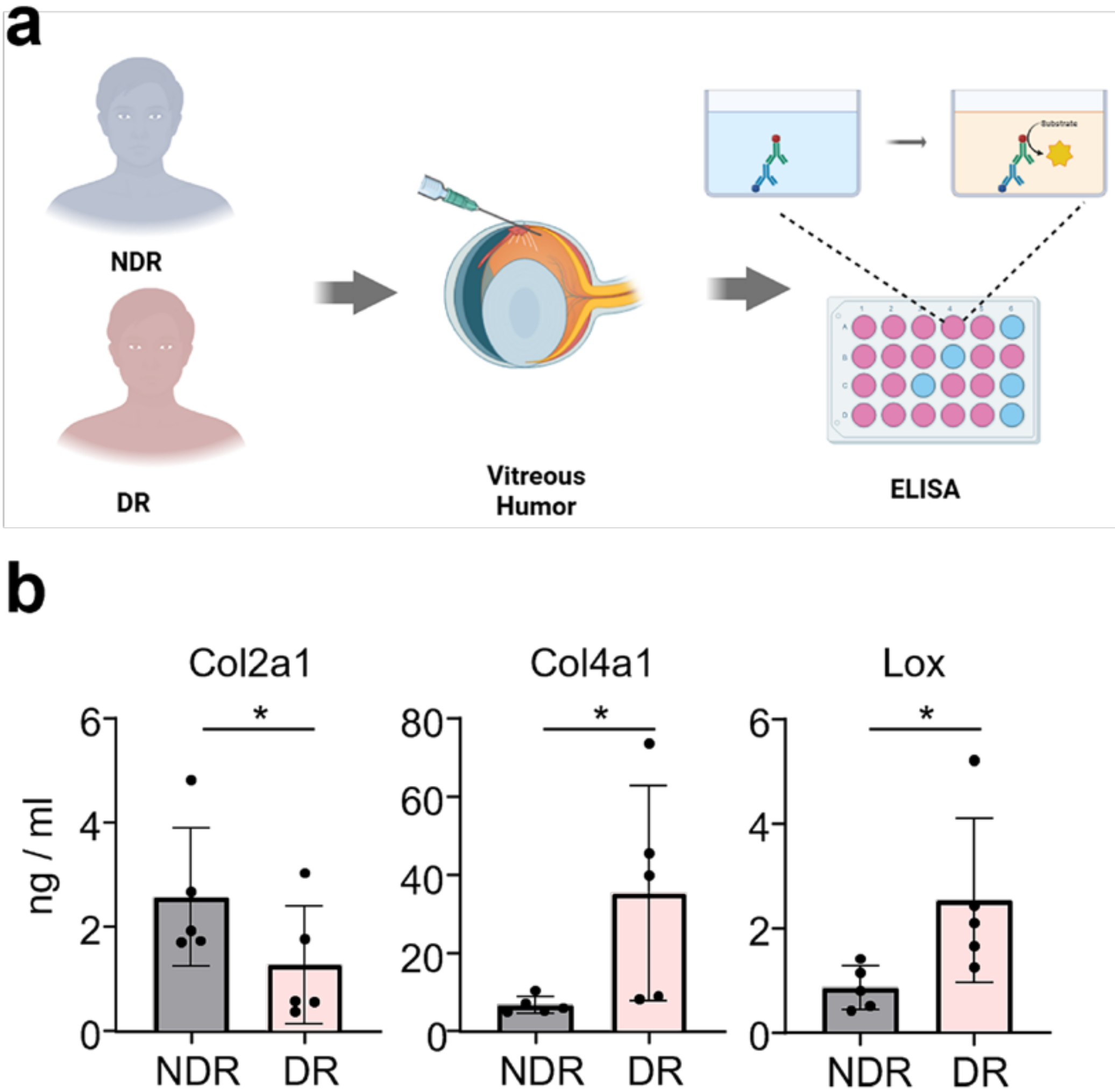

Supplementary Fig. 7. Collagens and Lox are elevated in DR patients.

(a) Schematic workflow for collecting and analyzing vitreous humor from clinical samples. **(b)** ELISA evaluation of Col2a1, Col4a1 and Lox levels in vitreous samples (n = 5). NDR: non-diabetic retinopathy; DR: diabetic retinopathy. Data are presented as mean ± SD. Statistical significance was determined by two-tailed Student's *t*-test (b). *, $p < 0.05$.

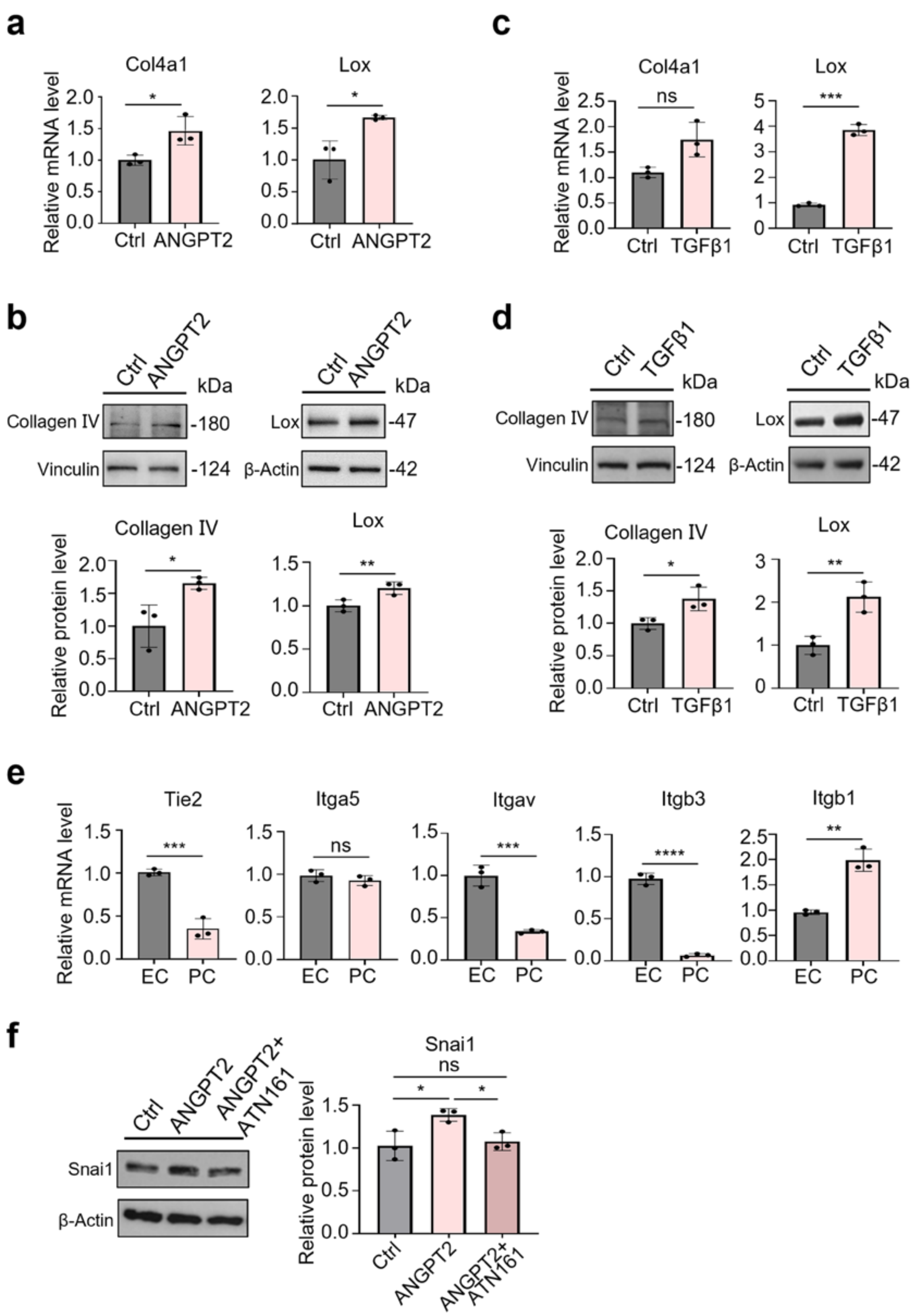

Supplementary Fig. 8. ANGPT2 and TGFβ1 promote Collagen IV and Lox expression in PCs.

(a and **b)** qRT-PCR (a) and western blotting (b) analysis of Collagen IV and Lox in ANGPT2-treated PCs (n = 3). **(c** and **d)** qRT-PCR (c) and western blotting (d) analysis of Collagen IV and Lox in TGFβ1-treated PCs (n = 3). **(e)** qRT-PCR analysis of ANGPT2 receptors Tie2, Itga5, Itgav, Itgb3 and Itgb1 between PCs and bEnd.3cells (n = 3). **(f)** Western blotting analysis of Snai1 in PCs following ANGPT2 stimulation with or without integrin α5β1 inhibitor (ATN161) (n = 3). Data are presented as mean ± SD. Statistical significance was determined by two-tailed Student's *t*-test (b). *, $p < 0.05$; **, $p < 0.01$; ***, $p < 0.001$; ****, $p < 0.0001$; ns, not significant.

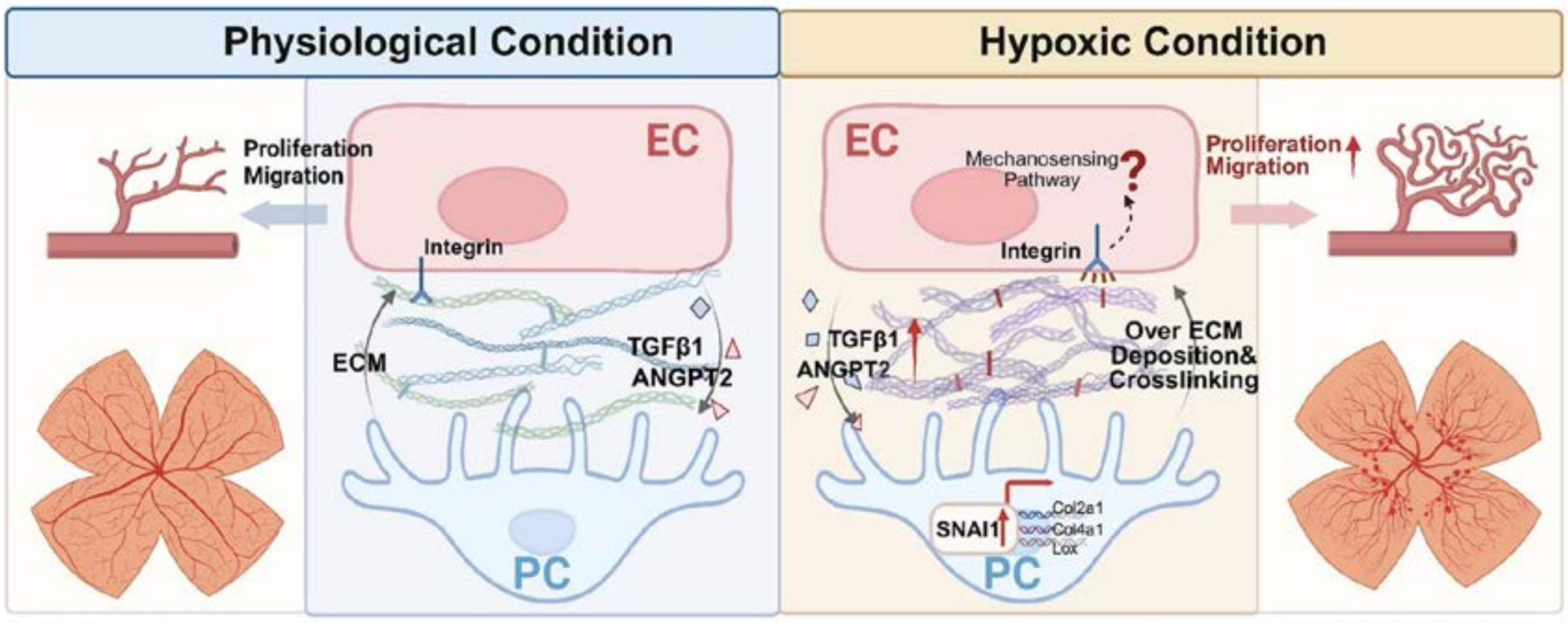

Supplementary Fig. 9. Schematic illustration of PC-EC crosstalk in normoxia and hypoxia.

Under normoxic conditions (**left**), retinal PCs maintain low basal expression of Snai1. Paracrine signaling between PCs and ECs preserves ECMs homeostasis, restraining excessive EC proliferation and migration to sustain vascular integrity. Upon hypoxic stress (**right**), EC-derived TGFβ1 and ANGPT2 synergistically drive Snai1 accumulation in PCs. Elevated Snai1 transcriptionally upregulates ECM-remodeling genes (e.g., Col4a1, Lox), promoting collagen deposition and crosslinking. This remodeled microenvironment in turn fuels EC proliferation and migration, culminating in disorganized pathological NV.

Supplementary Table 1. Information of patients involved in the study

No	Group	Sex	Specimen type	Diagnosis
1	DR	F	VH, FVM,	Bilateral VH + Bilateral DR + Left eye ERM
2	DR	M	VH	Right eye RD + Bilateral cataract
3	DR	F	VH	Right eye VH + Bilateral DR
4	DR	M	VH	Left eye VH + Bilateral DR
5	DR	M	VH, FVM, SRM	Bilateral VH + Bilateral RD + Bilateral EM + Bilateral DR
6	DR	F	FVM	Right eye VH
7	DR	M	FVM	Right eye VH
8	Ctrl	F	VH,	Right eye RD + Bilateral cataract
9	Ctrl	M	VH	Right eye RD + Bilateral cataract
10	Ctrl	F	VH	Left eye MH
11	Ctrl	F	VH	Left eye RD + Bilateral cataract
12	Ctrl	F	VH	Right eye RD + Right eye VH + Bilateral MH
13	Ctrl	M	SRM	Left eye RD
14	Ctrl	F	ERM	Left eye ERM

DR, diabetic retinopathy. F, female. VH, vitreous humor. FVM, fibrovascular membrane. VH, vitreous hemorrhage. ERM, epiretinal membrane. M, male. RD, retinal detachment. Ctrl, control. SRM, subretinal membrane. MH, macular hole.

Supplementary Table 2. Primers

Primer name	Forward (5'-3')	Reverse (5'-3')
Mouse Snai1 (qRT-PCR)	TGTCTGCACGACCTGTGGAAAG	CTTCACATCCGAGTGGGTTTGG
Mouse Rpl13a (qRT-PCR)	CTGCTCTCAAGGTTGTTCGGCT	CCTTCCGTTTCTCCTCCAGAGT
Mouse Acta2 (qRT-PCR)	TGCTGACAGAGGCACCACTGAA	CAGTTGTACGTCCAGAGGCATAG
Mouse Sm22 (qRT-PCR)	GCAGATGGAACAGGTGGCTCAA	CCCAAAGCCATTAGAGTCCTCTG
Mouse Col1a1 (qRT-PCR)	CCTCAGGGTATTGCTGGACAAC	CAGAAGGACCTTGTTTGCCAGG
Mouse Opn (qRT-PCR)	GCTTGGCTTATGGACTGAGGTC	CCTTAGACTCACCGCTCTTCATG
Mouse Mmp9 (qRT-PCR)	GCTGACTACGATAAGGACGGCA	TAGTGGTGCAGGCAGAGTAGGA
Mouse Mmp2 (qRT-PCR)	CAAGGATGGACTCCTGGCACAT	TACTCGCCATCAGCGTTCCCAT
Mouse Vim (qRT-PCR)	CGGAAAGTGGAATCCTTGCAGG	AGCAGTGAGGTCAGGCTTGGAA
Mouse Col4a1 (qRT-PCR)	ATGGCTTGCCTGGAGAGATAGG	TGGTTGCCCTTTGAGTCCTGGA
Mouse Col2a1 (qRT-PCR)	GCTGGTGAAGAAGGCAAACGAG	CCATCTTGACCTGGGAATCCAC
Mouse Lox (qRT-PCR)	CATCGGACTTCTTACCAAGCCG	GGCATCAAGCAGGTCATAGTGG
Mouse Plod (qRT-PCR)	CTGCCGAATCTTCCAGAACCTG	CCATTGCCATGAACGACCACTG
Mouse Col4a1 (ChIP negative control)	GGTGTCCCAAGCTGTGTCTA	CCAGTACCAGTGTGTGTGTGT
Mouse Col4a1 (ChIP Site1)	GACATTCTTCCCTTGCTGCT	CGTGTGCATTGTAGGTGTGT
Mouse Col4a1 (ChIP Site2)	GAAAGCGACCAAAACATGCG	CCTTTGCTAACCCGCTTTGT

Mouse Col2a1 (ChIP negative control)	GCAACACAGAAACCCACTTCG	CACCTACCTTCCTTGCTCCC
Mouse Col2a1 (ChIP Site)	CGGTTGCTCTATCCAGGCTA	CTGTGTGGAGGTCTAAGGGG
Mouse Lox (ChIP negative control)	ACCACACTACTAGGGGTCGG	TCTGCTGGTTTGATTGCTTCC
Mouse Lox (ChIP Site)	GGGGTGAGTGTGTATTGGGA	AGACTCAGACAGACGAAGGC
Mouse Tie2	GAACTGAGGACGCTTCCACATTC	TCAGAAACGCCAACAGCACGGT
Mouse Itga5	ACCTGGACCAAGACGGCTACAA	CTGGGAAGGTTTAGTGCTCAGTC
Mouse Itgav	GTGTGAGGAACTGGTCGCCTAT	CCGTTCTCTGGTCCAACCGATA
Mouse Itgb3	GTGAGTGCGATGACTTCTCCTG	CAGGTGTCAGTGCGTGTAGTAC
Mouse Itgb1	CTCCAGAAGGTGGCTTTGATGC	GTGAAACCCAGCATCCGTGGAA
Pdgfrb Cre (Genotype)	GTCACATACAGGGAAACTAAGACTCAT	CAGAAGCATTTTCCAGGTATGCTC
Snai1 flox (Genotype)	CGGGCTTAGGTGTTTTCAGA	CTTGCTTGGTACCTGCCTTC
Snai1 siRNA	GCATGTCCTTGCTCCACAA	GCAUGUCCUUGCUCCACAA
Col4a1 siRNA	GUGCAUGAGAAGAACAUAATT	UUAUGUUCUUCUCAUGCACTT

Supplementary Table 3. Antibodies

Product name	Company	Product code
Anti-PECAM1	RD	AF3628
Anti-PDGFRB	Invitrogen	14-1402-82
Anti-SNAI1 (IF)	Invitrogen	MA5-14801
Anti-SNAI1 (WB)	CST	C15D3
Alexa Fluor® 488 Anti-ERG	Abcam	ab196374
Anti-β-ACTIN	Proteintech	66009-1-Ig
Anti-α-SMA	Abcam	ab124964
Anti-GAPDH	Proteintech	60004-1-Ig
Anti-VINCULIN	Proteintech	26520-1-AP
Anti-NG2	Millipore	ab5320
Anti-KI67	Abcam	ab16667
Anti-COLLAGEN IV	Bio-Rad	2150-1470
Anti-LOX	Abcam	ab174316
Anti-FLAG	CST	14793S
HRP-anti-mouse IgG	CST	7076
HRP-anti-rabbit IgG	CST	7074
Alexa Fluor® 488-anti-goat IgG	Invitrogen	A11055
Alexa Fluor® 488-anti-rat IgG	Invitrogen	A21208
Alexa Fluor® 594-anti-rat IgG	Invitrogen	A21209
Alexa Fluor®594-anti-rabbit IgG	Invitrogen	A21207
Alexa Fluor®647-anti-rabbit IgG	Invitrogen	A32795

Supplementary Table 4. Quality control data for single-cell sequence analysis

Sample	P17	OIR P17-1	OIR P17-2-
Number of reads	278464342	332957394	278616741
Valid barcodes	96.4%	97.3%	94.9%
Sequencing saturation	53.3%	57.7%	73.5%
Q30 bases in barcode	95.0%	95.5%	95.9%
Q30 bases in RNA read	90.1%	89.5%	90.7%
Q30 in UMI	94.5%	95.3%	95%
Estimated number of cells	11486	8181	7614
Fraction reads in cells	47.5%	62.6^	50.8%
Mean reads per cell	24243	40698	36592
Total genes detected	786	817	701
Median UMI counts per cell	1268	1387	1145
Reads mapped confidently to genome	90.7%	88.5%	89.4%
Reads mapped confidently to intergenic regions	6.8%	7.2%	7.2%
Reads mapped confidently to intronic regions	34.2%	24.8%	33.7%
Reads mapped confidently to exonic regions	49.7%	56.5%	48.5%
Reads mapped confidently to transcriptome	43.8%	51.7%	41.6%
